# Managing hypertension in rural Uganda: Realities and strategies 10 years of experience at a district hospital chronic disease clinic

**DOI:** 10.1371/journal.pone.0234049

**Published:** 2020-06-05

**Authors:** Joseph H. Stephens, Faraz Alizadeh, John Bosco Bamwine, Michael Baganizi, Gloria Fung Chaw, Morgen Yao Cohen, Amit Patel, K. J. Schaefle, Jasdeep Singh Mangat, Joel Mukiza, Gerald A. Paccione

**Affiliations:** 1 Kisoro District Hospital, Kisoro, Uganda; 2 Doctors for Global Health, Kisoro, Uganda; 3 Albert Einstein College of Medicine/Montefiore Medical Center, New York, New York, United States of America; 4 Boston Children’s Hospital/Harvard Medical School, Boston, Massachusetts, United States of America; 5 Boston Medical Center/Boston University, Boston, Massachusetts, United States of America; RTI International, UNITED STATES

## Abstract

The literature on the global burden of noncommunicable diseases (NCDs) contrasts a spiraling epidemic centered in low-income countries with low levels of awareness, risk factor control, infrastructure, personnel and funding. There are few data-based reports of broad and interconnected strategies to address these challenges where they hit hardest. Kisoro district in Southwest Uganda is rural, remote, over-populated and poor, the majority of its population working as subsistence farmers. This paper describes the 10-year experience of a tri-partite collaboration between Kisoro District Hospital, a New York teaching hospital, and a US-based NGO delivering hypertension services to the district. Using data from patient and pharmacy registers and a random sample of charts reviewed manually, we describe both common and often-overlooked barriers to quality care (clinic overcrowding, drug stockouts, provider shortages, visit non-adherence, and uninformative medical records) and strategies adopted to address these barriers (locally-adapted treatment guidelines, patient-clinic-pharmacy cost sharing, appointment systems, workforce development, patient-provider continuity initiatives, and ongoing data monitoring). We find that: 1) although following CVD risk-based treatment guidelines could safely allocate scarce medications to the highest-risk patients first, national guidelines emphasizing treatment at blood pressures over 140/90 mmHg ignore the reality of “stockouts” and conflict with this goal; 2) often-overlooked barriers to quality care such as poor quality medical records, clinic disorganization and local employment practices are surmountable; 3) cost-sharing initiatives partially fill the gap during stockouts of government supplied medications, but still may be insufficient for the poorest patients; 4) frequent prolonged lapses in care may be the norm for most known hypertensives in rural SSA, and 5) ongoing data monitoring can identify local barriers to quality care and provide the impetus to ameliorate them. We anticipate that our 10-year experience adapting to the complex challenges of hypertension management and a granular description of the solutions we devised will be of benefit to others managing chronic disease in similar rural African communities.

## Introduction

The global burden of noncommunicable diseases (NCDs) is immense and growing. In 2012, NCDs, primarily cardiovascular diseases, cancer and chronic respiratory disease, were responsible for 68% of deaths worldwide with hypertension the largest modifiable risk for disease[1–3]. The highest prevalence of adult hypertension globally is in the WHO’s African region (35–38%), with the mean age of hypertensives younger than in the West—late 30’s to 40’s [4, 5]. In Sub-Saharan Africa (SSA), CVD is the leading cause of death among those older than 30 years, with stroke and hypertensive heart disease predominating [5, 6]. The economic burden of CVD in SSA, fueled principally by hypertension and the cost of caring for patients with its complications, is significant and rising rapidly [7].

In Uganda, as in the rest of SSA, paramount among the many barriers to effective NCD management are underfunding, workforce shortages, long wait times, provider knowledge deficits, poor infrastructure, lack of access to affordable medications, and expense of transport [5, 8–12]. These systems issues are compounded by patient conceptions of hypertension that affect health seeking behaviors [11]. In the Uganda 2014 national NCD survey, 70% of participants (ages 18–69) had never had their blood pressure measured. Of those with >30% 10-year risk of developing CVD, only 13% had been treated or counseled[13]. That same year, the Programme for the Prevention and Control of NCDs was allocated only 11.3% of the overall health budget, and 90% of that came from a 5-year grant from the World Diabetes Foundation. The national government directed only 0.011% of its own health budget to chronic disease [9]. A needs assessment performed in 13 Regional Referral Hospitals, 27 general (district) hospitals and 13 health Center IV’s in Uganda showed significant deficits in equipment, health infrastructure, clinical preparedness of providers and medication availability. Less than half of all health facilities had the essential screening equipment and tests for NCD risk factors. Only 18.5% of general hospitals had a hypertension clinic and only 11% had guidelines for treating hypertension accessible to staff. Perhaps most indicative of the inadequate infrastructure to care for NCDs, less than half of regional and general hospitals even kept patient files, and a similar number lacked an NCD patient register. Moreover, most non-M.D. providers had little confidence in managing NCDs. 75% of clinical officers (non-MDs similar to physician assistants) and 31% of medical officers (M.D. with 1 post-graduate year of generalist training) felt their clinical training did not adequately prepare them to manage hypertension [14].

Kisoro District in Southwestern Uganda has a population of 282,000–the majority (90%) living in rural villages and working as subsistence farmers (86%), earning 2 USD/day. Most women and a substantial minority of men are illiterate [15, 16]. Extrapolating from national rates of hypertension among adults 18–69 years old, an estimated 8–9,000 live in Kisoro with blood pressure (BP) above 160/100, representing 6.6–8.1% of the population. The high end of this range includes those on medication with a controlled BP [17]. While most patients in the district live within 5km of the nearest primary health center, resources for chronic disease are negligible at these primary facilities [8, 15]. Health Center IIIs, staffed by a nurse or clinical officer, receive deliveries of a thiazide diuretic and a calcium channel blocker (nifedipine) quarterly, but the quantity is inadequate (e.g. 200 tablets of nifedipine every 3 months), longitudinal records of hypertensive patients are not kept, and few patients seek chronic disease care at these facilities. In fact, a survey we conducted in early 2020 of 2 busy HC IIIs which refer to KDH, revealed that only 71 patients were given one or more prescriptions for BP drugs over a 3-month period.

To effectively manage hypertension in this rural population, we developed two programs which together care for over 1000 patients with hypertension: the Chronic Care Clinic (CCC) in the district hospital, and the Chronic Disease in the Community Program (CDCom), administered by local village health workers in the outlying villages. A previous paper by O’Neil, et al reported the efficacy of the CDCom program [18].

In this report, we describe challenges of managing hypertensive patients in Kisoro hospital’s CCC and the solutions we developed to address them over the course of a decade. We draw on data from our patient registry, charts, pharmacy records and quality improvement initiatives to define the problems and describe and evaluate the solutions. We expect that this data-informed description of novel responses to widespread challenges—developed in a non-research environment—will benefit others managing chronic disease in similar rural communities of SSA.

## Methods

### Description of study site and population

Kisoro District Hospital (KDH), the government hospital in the area and one of 2 hospitals overall, employs 2–3 medical officers and 6–8 clinical officers. These clinicians cover 142 inpatient beds (Ugandan average 125) in 4 clinical departments (medicine, surgery, pediatrics, and obstetrics-gynecology), admit over 9000 inpatients (40% more than Ugandan average), and see 50,000 outpatients (60% over Ugandan average) [19]. Since 2006, the busy but very understaffed hospital has collaborated with Doctors for Global Health (DGH), a US-community-based NGO, and Montefiore Hospital/Albert Einstein College of Medicine in New York, to provide internists for the hospital and support health activities in the villages. A team of one Ugandan medical officer and 2–3 U.S. PGY III Medicine residents staff the medicine wards of 50–70 beds 10–11 months per year, all supervised on the wards and in clinic by a Kisoro-experienced board-certified internist from the Global Health and Clinical Skills faculty of Montefiore. U.S. senior medical students are present 4–6 months/year.

In 2006, the tripartite collaboration established the Chronic Care Clinic (CCC)—the only publicly financed health facility in the district delivering continuous care to patients with hypertension, diabetes, heart disease, asthma, epilepsy and other NCD’s. Patients are referred from the wards after hospital discharge, and from private and lower-level public clinics. Laboratory and imaging resources are limited: X-rays and ultrasound are available about half the time, and echocardiogram is expensive and a 2-3-day journey away. In 2020, laboratory tests include CBC, malaria rapid-tests and smear, and tuberculosis GenXpert. Liver enzymes and creatinine (not available at KDH when these patient data were collected) are only intermittently available, while electrolytes, INR, and serologies are not available. Diagnoses for heart failure, asthma, and epilepsy are purely clinical. Chronic disease care was introduced and initially provided only by Western faculty and trainees, but, as will be discussed, has steadily evolved toward Ugandan providers. As of November 2017, when most of the data for this paper was collected, the CCC had approximately 800 active patients (63% female)—348 of whom were hypertensive—and saw an average of 50–60 patients per clinic, 3 times weekly. Presently in 2020, there are over 1400 active patients enrolled, nearly half with hypertension.

### Problems in hypertension management at KDH

The following obstacles impeding chronic disease care at KDH are presented according to the WHO Health Systems Building Blocks in brackets at the end of each heading:

#### 1) Provider turnover and knowledge about hypertension management [Health Workforce]

Lack of a stable workforce skilled in chronic disease management is the norm in rural Uganda (14), and Kisoro has been no exception. Prior to the establishment of the CCC, patients with chronic diseases admitted to the wards were treated acutely and sent home, inevitably to be readmitted weeks to months after their hospital medications ran out. Staff turnover is high and many levels of hospital administration control staff job-definitions. Staff can be redeployed by the district health officer, superintendent of the hospital, chief nursing officer and nurse “in-charge”–all of whom have varying priorities and change roles periodically themselves. Few are familiar with the training/expertise required for competent NCD management or the benefits of continuity of care.

In this context, the CCC was established by faculty from Montefiore Hospital/Albert Einstein College of Medicine in New York who were well-versed in chronic disease management and worked in primary care clinics that emphasized provider-patient continuity. However, the program has been unable to engage consistent *government-employed* staff as district hospital nurses and clinical officers regularly rotate deployments among departments. Thus, while the long-term goal of the CCC was always to develop *local* chronic disease expertise, until 2016 the primary clinician workforce were mostly rotating US medical residents and students supervised by US faculty.

#### 2) Drug stockouts and poverty [Access to Essential Medications/Service Delivery]

In 2001, the Ugandan government abolished user fees at public hospitals, and all services, including medications, became free to patients. However in Kisoro, as in most rural regions of Uganda, “stockouts” of the free drugs are common, and the majority of patients are too poor to afford routine purchase of medication for asymptomatic chronic disease [14]. Prior to every clinic, the CCC team checks with the hospital pharmacy and documents drug availability on a designated CCC medication form, copies of which are then distributed to each provider. The stockout data in Table 1 are the product of a 6-month retrospective analysis of these forms from July to December 2017.

**Table 1:** Drug availability: Percent of days in which hypertension medications at the CCC pharmacy were in-stock from July-December 2017.

Medication	Days in Stock	Medication	Days in Stock
Bendroflumethiazide	100%	Hydralazine	0%
Captopril	56%	Spironolactone	0%
Nifedipine	56%	Methyldopa	24%
Propranolol	47%	Atenolol	0%

While thiazides were always in-stock during this period, calcium channel blockers, ace-inhibitors and beta blockers were each in-stock only half the time. On 22% of clinic days, thiazides were the only first-line agent available. During stockouts, patients either payed full price for a month’s worth of medication from a local private pharmacy or, more often, went without.

#### 3) Overcrowding and patient-provider discontinuity [Service Delivery]

After 3 years of steady growth, the initial half-day clinic expanded to full-day, and after another year, opened its doors on a second day. Patients would be admonished by their providers to return at an approximate interval, usually a month, but patients often clocked that by pills remaining or their overall well-being. Without clinic appointments, they just showed up to be seen by whichever provider was available. Two years later, the waits were again intolerable. Patients would line up 1–2 hours before doors opened, by 9AM the wait would be 3 hours, and by 10AM often 4–5 hours. Including travel time to KDH, CCC became a monthly, all-day ritual. The situation was particularly detrimental for the sick or those that lived far away for whom getting to clinic was particularly taxing, the wait longest, and the return trip latest.

#### 4) Poor quality medical record keeping [Health Information Systems/Service Delivery]

While the CCC uses an electronic patient register with basic demographic measures for quality improvement initiatives, KDH is many years away from a smoothly functioning, confidential, easily understood and universally utilized electronic medical record system. Computers are scarce, staff untrained, local internet network absent, and power unreliable. So, paper is used: first stapled sheets and now charts bound by plastic.

But the real problem with the medical record was its *content*, not its cover. Most notes, even by U.S. trained residents, lacked appropriate clinical detail, documented key symptoms and signs inconsistently, and obscured the reasons decisions were made. Problems blurred together. As the number of visits per patient mounted and the clinic became busier, chart reviews became brief perusals of the last incomplete note, and patient care suffered. These twin problems of patient-provider discontinuity and lack of thoughtful documentation are nearly universal in the overcrowded and understaffed public clinics of Africa.

#### 5) Access to and adherence with care—Defining the problem of lapses from care [Service Delivery]

In order to fairly apportion a limited supply of medications (discussed below), patients are given a 30-day supply and an appointment to return in 1 month. Thus, a lapse in clinic attendance of more than 2 months strongly suggests either significant therapeutic non-adherence (since management of hypertension is rare outside of KDH-DGH chronic disease programs), or purchase of anti-hypertensive medication from a private pharmacy. The latter would be highly unusual for our impoverished patients, especially for an asymptomatic condition like hypertension. So, how frequently do patients with hypertension in rural Africa lapse from care, and for how long? The literature could not adequately answer this question despite its importance for program development and resource allocation.

#### 6) Patient knowledge about hypertension [Service Delivery]

What do rural Ugandan patients with little formal education think about the “silent” disease they’ve been told they have and how do their concepts influence their health behavior? To gain insight into these questions, identify misconceptions that could influence practice, and plan patient-centered educational strategies, we conducted confidential, verbal interviews with 2 groups of patients at opposite ends of the adherence spectrum: one of adherent patients waiting to be seen in the CCC, and the other, of patients who had dropped out of the CCC in 2016.

### Data analyzed

The data supporting our program assessment primarily come from a retrospective review of medical records involving all (N 348) adults enrolled in care for hypertension between January 2006 and Jan 2016. Upon first visit, all patients are given a unique medical record number to prevent duplication of charts. For the study, enrollment in care was defined as making at least two CCC visits in total, and at least one visit in the past year. More detailed assessments were conducted on a 139-patient subset of these 348 hypertensive patients through manual paper-chart audits. For these assessments, 150 patients were initially chosen randomly from 3 equivalent-sized cohorts of patients who enrolled in the CCC during different periods: 50 from 2006–2010, 50 from 2011–2013 and the final 50 from 2014–2015. 11 charts were missing, equally distributed between cohorts. As such 139 charts were reviewed.

Clinical data are supplemented by 2 quality improvement surveys of individual hypertensive patients to assess their beliefs and behavior regarding the condition. The first is a convenience sample of 100 patients in the clinic waiting room, identified from the appointment book in advance, and selected by random numbers to participate in the hypertension survey. All designated patients consented to the anonymous survey, and were interviewed privately for an average of 10–15 minutes by a trained interviewer prior to entering the waiting room. The other is of patients who dropped out of the CCC in 2016 (N = 58) and were deemed clinically eligible via severity criteria to be traced in the community and re-engaged in care by our program’s “Follow-up Project”[20]. Such patients are routinely surveyed about their knowledge, attitudes and practices around their disease, and offered short health talks to improve adherence with clinic appointments and medication.

We also present data from a retrospective review of a drug voucher initiative- a response to the medication stockouts described above. The data first describes voucher use over one year (2016), and then a follow up over 3 months (December 2019-February 2020) after adjustment of voucher subsidies according to self-described patient wealth.

To inform our understanding of adherence in hypertensive patients in rural Africa, a retrospective chart review was performed to delineate the frequency and natural history of lapses in care—defined as >3 months away from clinic. Two groups were assessed: an incidence group of 112 patients *newly enrolled* from May 2015 to April 2016, and a prevalence group of 248 patients active in the clinic as of 2015. Both groups were followed for one year, with follow-up through 2016.

### Ethics statement

Kisoro District Hospital and the Albert Einstein College of Medicine IRB approved this study (# 2017–8404).

## Results and solutions

### CCC patients

The 348 hypertensive patients enrolled in the CCC between 2006 and 2016, the subjects of this analysis, ranged from 21 to 93 years old (average 60), and were 65% female. 25% live in Kisoro town; the rest in surrounding villages. Patients live a mean (standard deviation-SD) of 8km (SD 10) from CCC and 16% travel 15km or more to clinic.

With differences likely dampened by the clinic’s high frequency of ward discharges, in comparison to their village-dwelling CCC counterparts, patients from town are more often overweight (39% vs 32%), obese (21 vs 15%) and diabetic (27 vs 20%). 5% of the patients have comorbid heart failure (CHF), primarily from hypertension, and fewer than 1% have been diagnosed with ischemic heart disease clinically. Of the 139 patients reviewed in detail (see below), 30 (22%) had a hypertensive complication recorded, but this is likely a marked underestimate, e.g. only 5 of the 139 ever had serum creatinine measured, and it was high in 2 of them. The recorded complications include clinically diagnosed stroke (9) or CHF (7), left ventricular hypertrophy (LVH) by EKG (9 of 43 EKGs), and nephropathy (7 had +2 to +4 dipstick-proteinuria or Cr>1.2 of 43 who received either test).

### Solutions to the problems in hypertension management at KDH

#### 1) Enhancing CD management capacity and knowledge, and overcoming high staff turnover [Health Workforce]

In the past 4 years, 4 local Kisoroans, 2 registered nurses and 2 clinical officers (herein referred to as *RN-COs*) have been trained and employed in the CCC. The RN-COs serve as primary providers, including medication initiation or alteration. Because their salaries are externally supported through DGH-Einstein *at the government rate*, they are not at risk of re-deployment or transfer. 3 of the 5 nurses (not RN-COs) in the CCC are employed by KDH, and 2 by DGH-Einstein. Of the 3 administrative staff, one is supported by KDH and 2 by DGH-Einstein. Precepting is available on-site by US-trained IM faculty, and strongly encouraged whenever patients have new symptoms, BP is seriously out of control or medication adjustment is contemplated. Though not “legally” required, adherence with this precepting guideline is high. The RN-COs see each CCC patient an average of 3–4 times a year and provide a critical measure of personal continuity.

Continuous learning is also promoted by 2 types of vignette writing and remote case-based learning with faculty in the US: the Kisoro trainees compose and submit ~8–12 challenging NCD cases per month, and synopses of CCC patients who died, to global health faculty in the US for analysis and feedback. The cases become the grist for 2 monthly “NCD seminars” and one “morbidity and mortality” discussion with the CCC providers that emphasize ambulatory management.

#### 2) Treatment strategy—Pragmatic and evidence-based; and responses to drug stockouts [Access to Essential Medications/Service Delivery]

*a) CCC treatment strategy*. To adapt to scarce resources, we’ve adopted a *risk-based therapeutic strategy* that maximizes cost-effectiveness by prioritizing CVD risk over absolute blood pressure thresholds [18]. Both approaches have advantages: numerical thresholds are easy to use, while, given the generally lower prevalence of atherogenic risk factors in this agrarian African community, a risk-based approach implies initiating hypertension treatment at higher levels of BP, treating fewer patients, and allocating drugs to those who’d benefit most.

The strategy employed in the CCC melds both approaches. The CCC has used the WHO CV risk prediction charts (2007) for “AFR E” region and treats those with a 20% or greater 10-year risk of CVD [21]. For example, patients 60 years and older with an SBP (systolic blood pressure) of >180 have at least a 20% risk, while those 40–60 years have a 10–20% risk. In practice, we were more conservative, choosing a lower threshold of treatment for all patients with uncomplicated hypertension of SBP >170 (measured at least twice on 2 separate clinic visits). An SBP of >170 places the non-diabetic patient in the top half of the 160–179 risk cohort—in a subgroup with a mean SBP of 175, and nevertheless a risk below 20%. The other exception to the “above 20%” rule is that all patients with stage 1 hypertension and either diabetes, kidney disease or complications of CVD are treated even if the 10-year risk is <20%. CCC does not treat non-diabetic patients with SBP less than 170 regardless of age or smoking status, as none of these patients have 10-year CVD risk greater than 20%. Once treatment is initiated for uncomplicated hypertension, the target SBP is ≤159, chosen as 10 points below the threshold of treatment.

**Fig 1:**
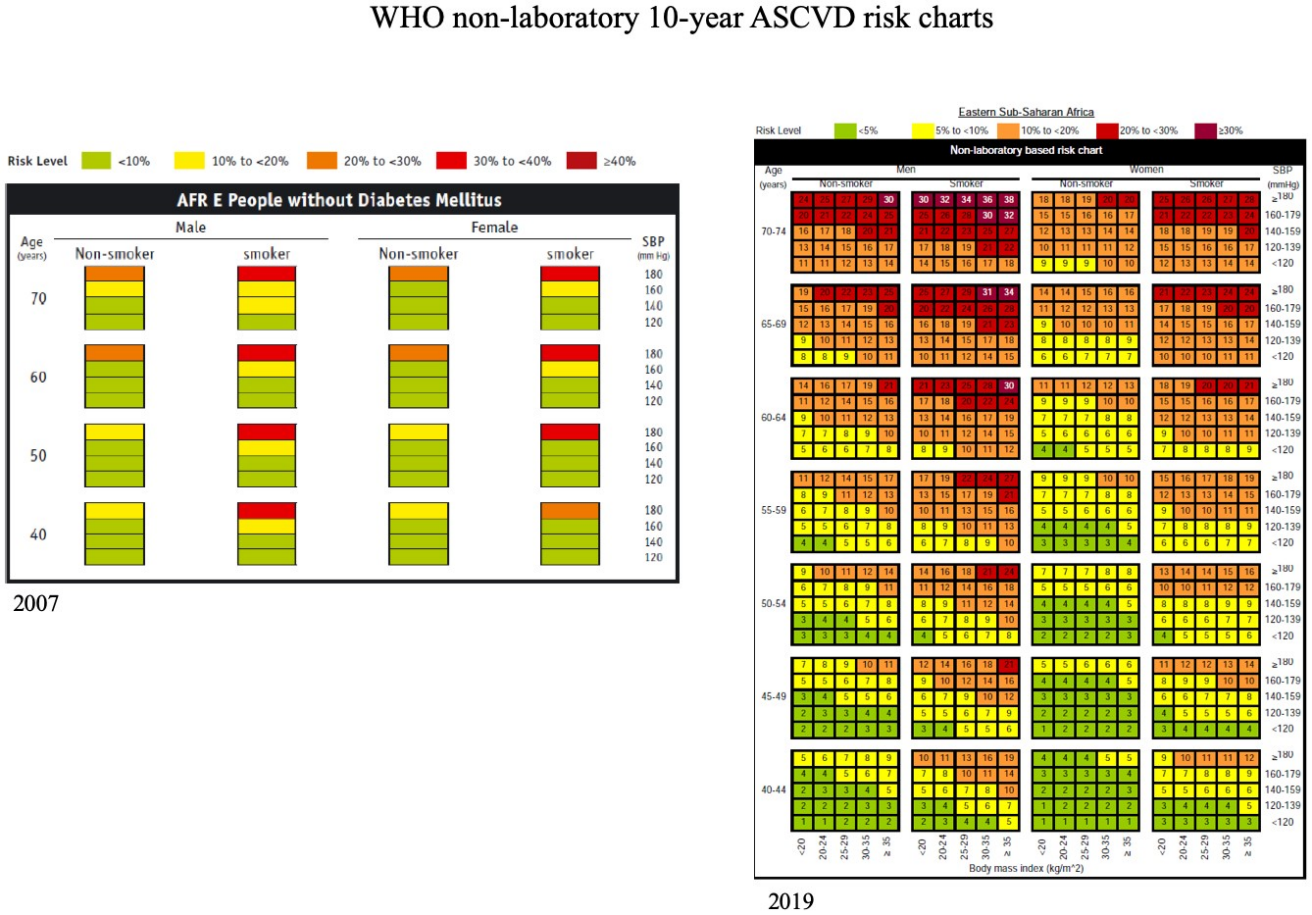
WHO CVD risk comparison charts for patients without diabetes. Left, 2007 “AFR E” field guidelines, stratified by Age, sex, SBP and smoking status. Right, 2019 “East SSA,” with further stratification by BMI.

In critically re-evaluating this treatment strategy 10 years later, we calculated the 10-year CVD event risk for all hypertensive patients without diabetes enrolled in the CCC using 2 different sets of CV risk estimates: the originally-employed WHO “AFR E” charts (which used age, smoking and SBP as variables, and death, myocardial infarction (MI) and stroke as CVD outcomes), and the newly published WHO 2019 non-laboratory CVD risk estimation guidelines[22]. The 2019 guidelines, which add BMI to the variables but maintain similar outcomes, are influenced heavily by progressive global urbanization with increasing CVD risk from sedentary lifestyle, obesity, dietary change, lipids, and stress at all BP levels (Fig 1). We applied each set of risk predictions individually to our non-diabetic CCC patients between 40–74 years, using a SBP cutoff of 170 mmHg. Of 281 patients without diabetes, 215 met criteria (66 were outside the eligible age range).

Using the 2007 guidelines, *none* of the CCC patients with BP >170 had a 10-year CVD risk of >20% and only 7% had a CVD risk >10%. The 2019 guidelines estimated 7% of patients had >20% risk and 53% had greater than 10% risk, with an average 10 year ASCVD risk for all patients with uncomplicated hypertension of 11%. Or, stated differently, using the updated 2019 guidelines *which likely exaggerates risk in our entirely rural population*, at our current treatment threshold of SBP >170 less than 10% of clinic patients with uncomplicated hypertension have a 10-year ASCVD risk >20.

*b) Responses to drug stockouts*. The frequency of drug stockouts has led to two additional less-than-ideal drug-management strategies, each with implications for equity and adherence. First, drugs are not prescribed to anyone for more than one month. Although requiring stable hypertensives to return to clinic monthly might negatively impact their adherence to treatment, provision of multi-month prescriptions to some would rapidly deplete the supply, and inadvertently allocate most of the drugs to the more clinically-stable patients.

Second, during stockouts, assistance is provided to patients to purchase drugs from the private sector. A cost-sharing “voucher plan” was developed with 3 partners: a local private pharmacy which discounts drugs by 20% in return for preferential referral of CCC patients, the NGO DGH, which covers an average of 40%, and the patient, who pays an average of 40%. From 2015-mid 2019, the percent contributed by each party was uniform: 20-40-40. Since mid-2019, depending on the wealth of the patient—as determined by a “wealth survey” conducted in the CCC of all 1400 patients over 4 months—DGH contributes 0–80% of the cost of the drugs, and the patient pays the balance. This has permitted full subsidy of the most indigent patients at no increased overall cost to DGH, an objective that was explained and deemed acceptable to the CCC patients. A CCC nurse writes the voucher when KDH meds have been exhausted; the patient, aware of exactly what s/he should pay, gives the voucher to the pharmacy when purchasing, and the pharmacy submits the vouchers to DGH for payment of its share at month’s end.

Between December 2019 and February 2020, 1763 drug vouchers were written, an average of 587 per month (range 556–637). Of these, 1043 (59%) were written for hypertension. Overall, patients filled 78% of the voucher prescriptions written, but the most indigent, who were *fully* subsidized through the voucher system, filled 85–90%. The average cost to DGH per voucher used during this period was 7303 USh, (or $2.00 USD). For the subset of hypertension patients, the average voucher per patient (all anti-hypertensives prescribed), was 5453 USh, or $1.50 USD. In 2016, an average of 246 CCC patients received vouchers every month, or less than half the present volume, while the average cost to DGH per voucher was $1.60 USD, or 25% less than at present. The increase per voucher over the past 4 years is due to increases in the price of essential drugs such as insulin, more frequent drug stockouts in the face of rising demand for CD medication, and an expansion in the number of important but more costly drugs we’ve opted to cover such as steroids. Of note, in 2016, only 67% of vouchers were filled, 10% fewer than now. This difference is partially due to the increment in voucher use by the indigent after wealth-based modifications were instituted.

#### 3) Addressing clinic overcrowding and patient-provider discontinuity through appointments and severity-of-disease criteria [Service Delivery]

To address the long waits and match patients to providers, an appointment system was adopted. Although appointment systems are rare in district health facilities in rural Uganda, and despite skepticism by some professionals that rural patients, who couldn’t read, would reliably return on an appointed day and hour, the appointment system has worked.

Since all return visits are in 1 month or less due to issues of drug availability discussed above, the focus of the CCC appointment system has less to do with the frequency of patient visits to the clinic, and more with *matching patients to provider-type and availability*. Based on “severity criteria,” complicated patients see MDs *every other visit* and for longer, while the less severe hypertensives see RN-COs, *for 10 of 12 visits a year*, for less time. Providers can also employ separate criteria for “stability”, and give “stable patients” simple “nurse refill visits”, skirting both MDs or RN-COs 1–2 times before seeing a provider again.

Our quality improvement data show that, when reliably enforced by CCC nurses, the system steadies patient flow throughout the day, allows providers more time with patients, and reduces patients’ time in clinic. The entire visit, from registration to departure with medications, remains less than 3 hours despite CCC membership having doubled between 2016 and 2020. Through the appointment system, each of the clinic’s RN-CO’s have their own patient panel, see their patients repeatedly over the year, and provide a critical measure of personal continuity to all patients in the clinic. (N.B. Accountability and consistency are important: when CCC intake staff failed to hold patients to their designated appointment times, clinic crowding temporarily returned).

#### 4) Concise and standardized medical records and flow sheets [Health Information Systems/Service Delivery]

To improve the usefulness of the chart and aid decision-making, we designed, and successfully implemented, “CCC forms” that highlight key clinical data, and flow-sheets to facilitate following patients with chronic disease.

While the initial-visit charting is “free-hand,” the forms, used for follow-up visits, have sections for the most common NCDs—hypertension, diabetes, CHF, asthma, epilepsy, and a generic “other”. In each section, disease-specific key features of *past* history appear on top, in bold—duration, severity measures, complications—followed by un-bolded *present* observations critical to decision-making—the last 3 blood pressure or glucose measures, recent medication changes, adherence, and targeted exam findings. If already documented in a prior note, the bolded past history items are simply *copied*. (The act of writing/copying helps internalize the content which reference to a distant, overlooked and outdated “problem list” does not, and takes little time.) The form permits less writing with more content and focus, facilitates decision-making by highlighting the relevant features of a chronic disease without needing to review the entire chart, and guides trainee case presentations.

To familiarize themselves with their patients’ histories, RN-COs have reviewed all charts in depth and filled out succinct flowsheets that summarize salient management decisions and outcomes over the years. Creating flowsheets has been an invaluable lesson in NCD care and facilitated accuracy and efficiency in clinic. [S1 and S2 Appendices]

#### 5) Access to and adherence with care—Defining the problem of lapses from care [Service Delivery]

Our analysis of adherence to clinic visits showed that, of the 112 hypertensive patients *newly enrolled* in 2015–2016, 36 (32%) lapsed; of these, 12 (33%) returned. Thus, nearly 80% of patients in their first year of enrollment either didn’t lapse or returned after a lapse, but of those who lapsed, most never returned.

Of 248 patients in the prevalence group, 141 (57%) had at least 1 lapse of 3 months in the year, but 129 (91%) of these returned, and 112 (91%) of the returnees were still in clinic a year later. The mean and median length of lapses for patients in the prevalence group was 5 months. Rates of lapsing were relatively similar between different severity groups. Of those without a significant lapse in care, 94% were in clinic a year later. The implications of these findings are discussed below.

#### 6) Patient knowledge about hypertension: Assessing patient beliefs [Service Delivery]

Data from the CCC survey of 100 hypertensive patients performed for quality improvement revealed that hypertension was largely (80%) conceptualized as a *symptomatic* illness, its genesis unknown. If untreated, nearly half thought it would lead to imminent death within an average of 8 months. However, largely due to provider insistence, many respondents (85%) recognized the need to take medications even when feeling well. Patients lost to follow-up saw it differently: when tracked in the community by our Follow-up Program (Alizadeh et al. [20]) and surveyed there, patients often cited feeling “better”–along with distance and clinic problems (overcrowding, and drug stocks outs)–as common reasons for clinic non-attendance.

#### 7) Blood pressure management results

Does the CCC succeed in lowering blood pressures? Given stockouts, lapses and turnovers, what clinical results are achieved? To address these questions, we turn to the analysis of the randomly selected 139 patients whose charts were manually reviewed in-depth.

At the time of their first CCC visit, 69 of the 139 patients (50%) were known hypertensives who had been prescribed antihypertensive medication; 70 were new diagnoses and treatment-naïve. The patients already on medication had an average (standard deviation) first-visit SBP of 157 mmHg (SD 24mmHg), and over their ensuing 2–10 years in the CCC with a mean of 17 recordings each, their mean SBP was 153 (SD 14). The 70 treatment-naïve patients had an average first-visit SBP of 178 mmHg (SD 28). Over the years, they also attained an average SBP of 153 mmHg (SD 14), a 25 mmHg drop. While the CCC targets a SBP ≤159, 41 patients (29%) had an average SBP over that goal, and 8 patients (5.7%) had an average SBP > 170 mmHg. The results were similar in the 27 diabetics reviewed: 26% averaged >159 while 3.7% (one patient) averaged >170. To achieve these results, the average patient was prescribed 1.6 antihypertensives. Of note, blood pressure regimens were adjusted on average every 4^th^ visit, or 2–3 times a year, often without documentation as to the reason for the change (58%), but frequently due to medication stock-outs (24%). Table 2 provides a synopsis of the obstacles, solutions, and recommendations in this report, organized according to pertinent WHO Health Systems Building Blocks.

**Table 2:** Summary of obstacles, solutions and recommended directions related to hypertension management in the CCC.

WHO Building Block Domain	Obstacle	Solutions	Directions Recommended
Health Workforce	-Provider knowledge	-Training mid-level providers (RN-CO’s) through precepting, case write-ups and NCD seminars	
Service Delivery/Access to Essential Medicines	-Frequent stock-outs and patient inability to pay for drugs	-CVD risk-based treatment strategy to avoid dispensing drugs to low risk patients to avoid harm, and re-direct supply to high risk patients to maximize benefit - Increase access to essential drugs; and when unable, cost-share through discounted medication vouchers between preferred pharmacies, patients, and non-government donors—increase support of subset of patients unable to afford vouchers	-Validate CVD field risk calculators with local data, or data from similar demographics -Improve government supply chain
Health Workforce	-Inconsistent, rotating provider deployments disrupt continuity, increase inefficiency	- Support for stable, non-rotating positions for NCD care	-Government-funded positions for continuous NCD providers
Service Delivery (Access/Efficiency/ Quality)	-Clinic overcrowding	- Appointment system - Dedicated RN-CO patient panels -Severity criteria to guide frequency of MD visits	-Expand to village-based care by supervised CHW’s
	-Patient-provider discontinuity		
	-Poor quality medical records	- Standard visit forms per disease - Flowsheets for key clinical information	-EHR with relevant clinical prompts, without copy-paste capacity
	-Patient understanding of hypertension	-Assessment of Patient Health Beliefs	-Patient Education Initiatives
Service Delivery (Coverage)/ Health Information Systems	-Community health and screening data divorced from clinical care -Inability of public system to support care for stage 1 hypertensives without other risk factors	-Increase awareness through community screening* - Expand NCD care through *supervised* CHWs*	-Train mobile supervision team to oversee community-based care - increase funding for medications to deliver at the community level
Health Information Systems	-Poor quality medical records	-Implement record forms that prompt documentation of key clinical data - Continuous clinic-wide data collection, with periodic evaluation	

CHW, Community Health Worker; NCD, non-communicable disease.

*These initiatives, currently underway in the CDCom initiative, will be described in a forthcoming manuscript.

## Discussion

This paper offers a data-informed, granular account of one district hospital’s attempt to manage hypertension in rural Africa—a mosaic of the challenges faced, strategies implemented, and results achieved in a typical region with few resources.

The report makes 3 principle contributions to our understanding of hypertension management in this setting:

### 1. Barriers to care: Supporting what’s known, and defining the devil’s details

Barriers to quality care involving patients, providers and hospital systems affect every aspect of our experience and permeate these pages. The sheer poverty of families and regional governments underpin most of these barriers. In sub-Saharan Africa, out of pocket costs for health care average 65% of all health expenditures[23], and some studies have reported as much as 10–30% of household income being spent on preventive care [24, 25]. The cost of transport often surpasses the cost of medication, and, with CCC patients living a median of 8 km from KDH and 16% living >15km away, most sacrifice 2 days’ wages to keep an appointment by taxi-motorcycle. A third of our patients could not afford even the 2/3 subsidy off the retail price available through the CCC vouchers.

Our experience and data support previously described impediments to hypertension control:

- patient (and often, provider) misunderstanding about hypertension, particularly its mythical connection to everyday symptoms and stress which affects adherence to therapy. 80% of our waiting-room clinic patients say they experience symptoms when their BP is elevated, and a third of those who ceased coming altogether thought they were cured;
- shortage of rural providers, who have little time for non-acute care and lack of training (or often interest) in chronic disease management. Kisoro district has a doctor-patient ratio of 1/20-40,000 versus 1/300 in the West;
- paucity of laboratory or imaging resources to detect complications or other CVD risks, e.g. since reagents were generally unavailable, only 3% of our CCC hypertensives had a creatinine documented;
- drug “stock-outs”: in the KDH pharmacy, drugs other than thiazides are missing half the time.

However, our experience also highlights other barriers to effective care that are more interstitial, often overlooked, and potentially remediable locally at low cost. These include:

- medical records, marked by inconsistent and sparse documentation of relevant findings, little clarity about what, how and why actions were taken, and poor continuity of care;
- clinic disorganization and over-crowding leading to patient loss of time, money, satisfaction, and adherence for an asymptomatic condition with serious, but distant, morbidity or mortality;
- frequent provider and support staff turnover, engrained in the employment practices of district hospitals and implemented by various leaders with differing priorities and little appreciation for the complexity of chronic disease management or the efficiency and cost-saving potential of patient-provider continuity. Training and continuity of care are casualties of the labor culture.

### 2. Treatment guidelines: Simplified, population-sensitive, and risk-based

We modified and employ simplified risk-based treatment guidelines that result in fewer patients being treated. To do this, we assumed population risk factor profiles consistent with our lean, non-smoking, physically active agrarian population, using the WHO “AFR E” risk tables. For easy adoption by low-resource clinics, we then converted these risk categories, that use multiple variables, into (higher) *blood pressure cut-offs* that vary only with clinically obvious complications and diabetes. We re-evaluated and confirmed the low predicted risk of this simplified approach, treating at a cutoff of ~170 SBP, in all 215 age-eligible clinic patients with hypertension, using both 2007 charts and updated 2019 charts. While the 2019 charts showed some increment in risk over the 2007 charts, likely due to the urbanization occurring across East Africa (but not yet in rural Kisoro), the calculated risk for almost all patients in the CCC with SBPs of 170 remained less than 20%.

The 170 cutoff in the CCC represents a significant departure from the Ugandan government guidelines (i.e. prompt treatment above 160, and after lifestyle modification is attempted for those between 140–160, Table 3) [26]. Although the 170 cut off has been largely motivated by a desire to treat first those most at-risk in the face of frequent drug stockouts, and to avoid inevitable out-of-pocket expenses that accompany medical diagnoses in a very poor population (travel to clinic, missed work, drugs), the results of the above risk analysis attest to the *safety* of our treatment approach. Indeed, given the national distribution of SBP, lowering our treatment threshold to SBP 160 would effectively *double* the proportion of patients with uncomplicated hypertension meeting criteria for treatment [17]. While the CCC has made great strides in improving provider workforce and increasing access to medications, lowering the treatment threshold would likely overwhelm staff, lengthen stockout periods, and shift affordable medications to primarily low risk individuals. Table 3 summarizes the CCC treatment approach alongside the WHO HEARTS and Uganda Ministry of Health 2012 Guidelines for comparison.

**Table 3:** WHO HEARTS Technical Package, Uganda Ministry of Health and Kisoro CCC CVD treatment comparisons.

WHO HEARTS Technical Package Domain	Ugandan Ministry of Health 2012 Guidelines	Relevant CCC initiative
**Healthy Lifestyle**	**Recommend**: No added salt, increase physical activity/exercise, reduce body weight, stop smoking, decrease alcohol intake.	**Recommend**: Same, although in our agrarian population, most lifestyle initiatives do not apply. Patient Health Beliefs Survey to inform educational initiatives
**Evidence Based Treatment Protocol**: -Risk <20%—counseling only, follow up every 3 months until risk <10% -Risk 20–30%- treat BP >140/90 -Risk >30%—treat BP’s over 130/80 and give a statin -All patients with DM—treat BP’s >130/80, give statin if over 40 -Treat all patients with established CVD, albuminuria, retinopathy, LVH -Treat all patients with BP >160/100 -Statin if cholesterol over 320mg/dl	**Recommend BP cut-points for treatment**: -BP >140/90—initial lifestyle counseling followed by antihypertensive administration if BP still > 140/90 at 3 month follow up. -BP >160/100—initiate treatment immediately.	**Risk Based Treatment protocol with absolute blood pressure thresholds**: -SBP >170 (on 2 readings at different visits)—initiate antihypertensive -All patients with DM, established CVD, kidney disease, LVH—treat SBP >140 with antihypertensive *Above protocol treats >90% of those with >20% risk according to WHO 2019 non-laboratory-based risk assessment charts for “East SSA” and *all* with >20% risk by WHO 2007 charts. 2007 risk predictions are likely to be more accurate for rural areas like Kisoro.
**Access to Essential Medicines and Technology**	**Meeting goals hampered by**: -Frequent stock outs of essential medications -Statins not on formulary	-Medication Voucher System during drug stock outs
**Risk Based Management**: 2019 WHO CVD risk assessment charts	-No recommendations regarding CVD risk assessment	-Validation of a modified risk-based treatment approach using WHO 2007 and 2019 guidelines
**Team Care And Task Sharing**		-Training RN-CO’s as primary providers
**Systems for Monitoring**		-Continuous data driven QI (patient registry, patient surveys, program monitoring)

DM, diabetes mellitus; LVH, left ventricular hypertrophy; QI, quality improvement

Two years ago, Kisoro was upgraded from a town to a small city mirroring the predominant form of urban expansion in Uganda: rural towns and small cities are growing more rapidly than large metropolitan centers. Thus, district hospital outpatient clinics in rural areas will care increasingly for “mixed” cohorts of hypertensives, one from surrounding villages with a relatively static, low prevalence of usually-undetected hypertension—lean, active, non-smoking and unable to afford or access junk food; and the other, townsfolk consuming globalization, the soldiers of modernity’s epidemiologic transition—sedentary, stressed, and surrounded by sugar. The first, low ischemic risk; the second, high, with risk factors often hidden from view. Risk scores, contextualized for Africa’s evolving rural-urban sub-regions and ideally incorporating patient sojourns through them over a lifetime, could add significantly to our ability to predict risk in remote regions of Africa. It would help define when high blood pressure becomes a “disease” in various locales, and at what cost. Finally, new research into “benefit-based” treatment also has significant implications for ASCVD prevention strategies, but more data is needed prior to its adoption for the treatment of hypertension [27].

### 3. Defining challenges and developing solutions

Real-life problems in hypertension management are addressed by novel solutions. Four themes stand out: the continuous use of local data to inform clinic practice, the “natural history” of adherence with care, innovative strategies to overcome barriers, and the blood pressures achieved with higher treatment thresholds in a rural district hospital short on medication.

#### a) Local data to inform practice

Since its inception, the larger KDH-DGH program has collected local data on all aspects of its work, either continuously or periodically, and uses such data to measure and solve problems (e.g. drug stockouts and creation of vouchers), facilitate patient education (e.g. hypertension knowledge, attitudes and practices survey), and evaluate and modify policies and interventions (e.g. setting feasible and safe BP targets). In every situation, the data are gathered by an extension of the work of already-employed staff, and the “research” cost has been modest.

#### b) Natural history of adherence with clinic

Lapses from clinic and presumably from treatment, are frequent, albeit less so among newly enrolled patients who may have had symptoms that recently brought them to medical attention or that heightened concern about their new diagnosis. If and when they do lapse from clinic however, most newly enrolled patients do not return—perhaps an expression of latent denial as they come to grips with having a chronic disease, or simply inability to adapt to constraints of the CCC. Contrariwise, a remarkable 57% of the long-term hypertensives lapsed for >3 months (average 5 months) over the year, but nearly all returned and remained in the clinic. Psychologically adapted to having hypertension but not affected by complications yet, they’ve relaxed. They’re survivors of both hypertension and the clinic. They believe their hypertension is significant—thus they return. But they also have “broken the rules” before and survived. So, they’ll work in the field today, and stretch their drugs over yet another month, again. (Although some may have accessed drugs elsewhere, as discussed previously these are likely to be very few, (<10%)).

#### c) Strategies to confront structural barriers to care: Prescription intervals and vouchers, appointments, staff, and forms

Naturally, lapses from the CCC are, in part, a response to the clinic’s shortages of medication and staff, which the clinic has addressed in myriad ways.

*Drug prescriptions and vouchers*. Stock-outs have required informal “rationing” of government-supplied free medication. The CCC thus limits prescriptions to one-month, which promotes equity at the cost of adherence, and utilizes a *pharmacy-voucher system*. The voucher system is a feasible *partial* solution to drug stock-outs that distributes the cost among its 3 beneficiaries: the patient, the pharmacy and a committed 3^rd^ party, in this case DGH/Einstein whose objective is NCD care for the indigent when the government cannot supply it. That a third of patients could not afford the prior 40% co-pay attests to the poverty in the district. Although only in place 8 months, modifying voucher amounts by the patient’s relative resources increased overall voucher use and fully subsidized drugs for the poor at no increase in total cost to the program.

Of course, the voucher system is but a half-way measure, and fails to address the root problem of inadequate government funding for both district hospitals and treatment of chronic disease [28]. Progress managing hypertension will need more than a verbal commitment from governments to care for their majority poor. Required are a robust investment in local public hospitals, and a consistent medication supply for chronic disease.

*Appointments*. A CCC *appointment system* matches patient load to provider availability depending on case complexity. Appointment systems are feasible, but require sensible, flexible guidelines implemented consistently by staff.

*Staff*. Through the creation of relatively *permanent* positions for local RN/CO’s, on-the-job training, and appointments, it is possible to develop a local workforce that delivers *personalized continuity care* to NCD patients. Although external funds were used to ensure position stability, salaries are equivalent to government salaries, and feasible.

*Medical record forms*. Tailored specifically for each patient and her co-morbidities, medical record forms for chronic diseases solicit key data necessary for quality care, and improve communication, continuity and reporting.

#### d) Blood Pressure management and control achieved: Better data needed

Our data suggest that despite lapses, stockouts, provider turnover and a higher treatment threshold of 170 SBP, the average CCC patient maintains an SBP of 150–155, 25 points lower than at enrolment. In a population with a low prevalence of other CV risks whose major potential complication is stroke, this is a gratifying change. However, the patient charts that yielded these results, although proportionate to and representative of sequential cohorts of clinic enrollees still active in the CCC, do not reflect an *inception cohort* of newly diagnosed hypertensives but rather “the survivors” with probably fewer complications and hypertension more amenable to control. Missing are an untold number who enrolled with them but dropped out due to death, severe morbidity, or simply non-adherence. Thus, the results are undoubtedly biased toward success, as are most reports from rural lower income countries that lacked the funding to enroll and follow “all-comers” from the beginning. We need better data.

## Limitations

Besides the biases of its retrospective data analyses mentioned above, many limitations of this report are inherent in the unique elements of the CCC, and actually a by-product of our collaboration. The CCC is a “teaching clinic” for both Americans and Ugandans, and many of its health providers are Western. Provider time spent per patient is greater than the usual district hospital outpatient visit (a plus) but could be perceived as slowing things down (a minus), and teaching clinics are not the norm in rural district hospitals. Other programmatic features of the CCC that potentially influence the outcomes reported include the clinic’s one-month return visit interval to facilitate equity in drug distribution, and the external support from DGH for pharmacy vouchers, training, and salaries. Describing the rationale and function of some of these special characteristics of the CCC is part of the objective of this report, but generalizations must be made with care. If elements of our experience are adopted elsewhere, they should be carefully chosen and adapted to local realities.

Lastly, streamlining the function of district hospital-based clinics like the CCC should be but a small part of the response to the emerging epidemic of cardiovascular disease in Africa. For maximal population impact, emphasis must be put on community engagement, and funding systems that shift screening, treatment and monitoring of hypertension to supervised community health workers.

## Supporting information

S1 Appendix Example CCC chart form. (PDF)

S2 Appendix Example CCC flow sheet. (DOCX)

S1 File (PDF)

S2 File (PDF)
