# Supplementary material for: Managing hypertension in rural Uganda: Realities and strategies 10 years of experience at a district hospital chronic disease clinic
Source: PLoS One. 2020 Jun 5;15(6):e0234049. doi: 10.1371/journal.pone.0234049 (PMC7274420; doi:10.1371/journal.pone.0234049)
Supplement: S1 Appendix — (PDF) [file pone.0234049.s001.pdf]

Chart number ____________ Name: ____________________________________ Sex: ________ Age: _______

A PAGE 2

Visit Date: ________________ Weight: ________ Triage BP (1) ___________ (2) _____________ Pulse _______
Time: _________ Blood sugar _____________ [ ] Fasting [ ] Random Time of last meal: ____________

Diabetes

Year dx'ed*:____________; *range* of past RBS**: _______ - _______ ***Complications: eye, neuro, renal, vascular, DKA, hospitalizations, (specify, ?severity):

Blood sugar x last 3 visits: ***(last)***________; _______; ______;
MED Δ's past 2 mos: ____ Adherence: ____________________;
Recent Hypo- or hyper-glycemia sxs (describe) AND Other DM-related sx's: ______________________________

P.E. BP x 2: __________; ___________; other notable (e.g. eye, neuro, etc.) ______________________________

Assessment, Rationale for Rx.changes: ____

HTN

***Year dx'ed:_______*; r*ange past* BP**: SBP______-_______ DBP______-______
Complications: (CHF, CVA, renal, etc) (specify; indicate *severity**; and **also** complete OTHER section below)***: ______________________

BP x last 3 visits: ***(last)***____________; ____________ ; ___________;
MED Δ's past 2 mos (specify): ___________________________________ Adherence :____________________;
PE: BP 1 _____ ___; BP 2____ ____ HT-related findings:______________________________; Other PE: ____________________
Assessment, Rationale for Rx.changes: ____

Cardiac

Problem (CHF, arrhythmia, etc) ___________________________; ***Etiology specify***: __________________________; ***Year dx'd:*** ______;
Hospitalizations *past year*? (dates, pbm, Rx):____:
MED Δ's past 2 mos: ____; Adherence:___________________;
Symptoms *now* (function, DOE orthopnea, PND); specify; note *CHANGES* ______________________________

P Exam (quantify) : Wt Δ________/time_______; BP__________; HR________; JVP______cm>angle Louis; HJR: ______;
Pulm dullness/rales: ________________________; ascites (how detected)/liver________; edema__________
Cardiac: ____
Assessment, Rationale for Rx.changes: ____

OTHER (*HIV, CVA,*

Problem/Diagnosis:* ___________________; *Main (past) symptoms*:** _____________________; ***Since when? ________;
HPI: Hx/Sx's/Rx: (specify, ?function, note CHANGES):____

Relevant PE: ____
Assessment, Rationale for Rx.changes: ____

MORE ROOM: for OTHER CO-MORBID diseases, NEW COMPLAINTS that don't "fit" in the above CD boxes, or ELABORATION on reasoning/plans

TEST results TODAY (N.B. also record on front Test sheet) ____
PRESENT Meds/Dose; **Adherence [**A) Taking as directed B) Taking differently C) Ran out D) O/S last visit**]; PLAN:** A) Continue; B) Dose Δ; xx) STOP
1.____________________________: A-D_____; Plan: A, B, XX; ____; 3. ___________________________: A-D___; Plan: A, B, XX; ____;
2. ___________________________: A-D_____; Plan: A, B, XX;____ ; 4. ___________________________: A-D___; Plan: A, B, XX; ____;
NEW MEDS/Dose and NEW DOSE CHANGES (of Old meds above):
1. _______________________________________; 2. _______________________________________; **CLINICIAN** (name): ________________
3. _______________________________________; 4. _______________________________________; **PRECEPTOR:** ___________________
