# Supplementary material for: Managing hypertension in rural Uganda: Realities and strategies 10 years of experience at a district hospital chronic disease clinic
Source: PLoS One. 2020 Jun 5;15(6):e0234049. doi: 10.1371/journal.pone.0234049 (PMC7274420; doi:10.1371/journal.pone.0234049)
Supplement: S2 Appendix — (DOCX) [file pone.0234049.s002.docx]

NAME: _____________________________ AGE:_________ SEX:______ FILE NUMBER:________ SEVERITY:______

| **DATE**  **[*oldest on top*]**  **CCC VISIT**  **or**  **ADMISSION**  ***(specify)*** | **GLUCOSE**  ***(indicate***  ***hours to***  ***last meal)*** | **BPs x 3** | **LAB or**  **IMAGING**  ***New Results*** | **MEDS/doses** | **ADHER-**  **ENCE?**  ***[overall]*** | **COMMENTS**  ***[e.g. symptoms, med or***  ***adherence notes, etc.]***  ***positive symptoms important to**  **Indicate for cardiac patients*** | **MED CHANGES** |
| --- | --- | --- | --- | --- | --- | --- | --- |
|  |  | **HR** |  |  |  |  |  |
|  |  | **WT** |  |  |  |  |  |
|  |  |  |  |  |  |  |  |
|  |  |  |  |  |  |  |  |
|  |  |  |  |  |  |  |  |
|  |  |  |  |  |  |  |  |
|  |  |  |  |  |  |  |  |
|  |  |  |  |  |  |  |  |
|  |  |  |  |  |  |  |  |
|  |  |  |  |  |  |  |  |
|  |  |  |  |  |  |  |  |
|  |  |  |  |  |  |  |  |
|  |  |  |  |  |  |  |  |
|  |  |  |  |  |  |  |  |
|  |  |  |  |  |  |  |  |
|  |  |  |  |  |  |  |  |
|  |  |  |  |  |  |  |  |
|  |  |  |  |  |  |  |  |
|  |  |  |  |  |  |  |  |
|  |  |  |  |  |  |  |  |
|  |  |  |  |  |  |  |  |
|  |  |  |  |  |  |  |  |
|  |  |  |  |  |  |  |  |
|  |  |  |  |  |  |  |  |
|  |  |  |  |  |  |  |  |
|  |  |  |  |  |  |  |  |
|  |  |  |  |  |  |  |  |
|  |  |  |  |  |  |  |  |
|  |  |  |  |  |  |  |  |
|  |  |  |  |  |  |  |  |
|  |  |  |  |  |  |  |  |
|  |  |  |  |  |  |  |  |
|  |  |  |  |  |  |  |  |
|  |  |  |  |  |  |  |  |
|  |  |  |  |  |  |  |  |
|  |  |  |  |  |  |  |  |
|  |  |  |  |  |  |  |  |
|  |  |  |  |  |  |  |  |
|  |  |  |  |  |  |  |  |
|  |  |  |  |  |  |  |  |
|  |  |  |  |  |  |  |  |
|  |  |  |  |  |  |  |  |
