# Supplementary material for: Managing hypertension in rural Uganda: Realities and strategies 10 years of experience at a district hospital chronic disease clinic
Source: PLoS One. 2020 Jun 5;15(6):e0234049. doi: 10.1371/journal.pone.0234049 (PMC7274420; doi:10.1371/journal.pone.0234049)
Supplement: S1 File — (PDF) [file pone.0234049.s003.pdf]

World Health Organization

Prevention of Cardiovascular Disease

Pocket Guidelines for Assessment and Management of Cardiovascular Risk

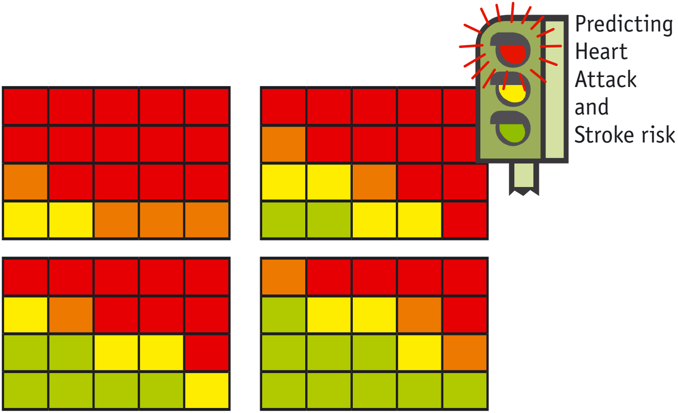

World Health Organization

Prevention of Cardiovascular Disease

Pocket Guidelines for Assessment and Management of Cardiovascular Risk

(WHO/ISH Cardiovascular Risk Prediction Charts
for WHO epidemiological sub-regions **AFR D and AFR E**)

Geneva, 2007

WHO library Cataloguing-in-Publication Data

Prevention of cardiovascular disease : pocket guidelines for assessment and management of cardiovascular risk : (WHO/ISH cardiovascular risk prediction charts for the African Region).

1.Cardiovascular diseases – prevention and control. 2.Cardiovascular diseases – diagnosis. 3.Risk factors. 4.Risk assessment. 5.Guidelines. 6.Charts. 7.African Region (WHO). I.World Health Organization. II.Title: Pocket guidelines for assessment and management of cardiovascular risk : (WHO/ISH cardiovascular risk prediction charts for the African Region).

ISBN 978 92 4 154726 0 (NLM classification: WG 120)

The risk prediction charts provided in these guidelines can be used only in the following countries:

AFR D sub-region: Algeria, Angola, Benin, Burkina Faso, Cameroon, Cape Verde, Chad, Comoros, Equatorial Guinea, Gabon, Gambia, Ghana, Guinea, Guinea-Bissau, Liberia, Madagascar, Mali, Mauritania, Mauritius, Niger, Nigeria, Sao Tome and Principe, Senegal, Seychelles, Sierra Leone, Togo

AFR E sub-region: Botswana, Burundi, Central African Republic, Congo, Côte d'Ivoire, Democratic Republic of the Congo, Eritrea, Ethiopia, Kenya, Lesotho, Malawi, Mozambique, Namibia, Rwanda, South Africa, Swaziland, Uganda, United Republic of Tanzania, Zambia, Zimbabwe

WHO/ISH charts for all WHO Member States are available on compact disc from WHO.

Other risk prediction charts are already available for Australia, Canada, New Zealand, the United States of America and many countries in Europe.

© World Health Organization 2007

All rights reserved. Publications of the World Health Organization can be obtained from WHO Press, World Health Organization, 20 Avenue Appia, 1211 Geneva 27, Switzerland (tel.: +41 22 791 3264; fax: +41 22 791 4857; e-mail: bookorders@who.int). Requests for permission to reproduce or translate WHO publications – whether for sale or for noncommercial distribution – should be addressed to WHO Press, at the above address (fax: +41 22 791 4806; e-mail: permissions@who.int).

The designations employed and the presentation of the material in this publication do not imply the expression of any opinion whatsoever on the part of the World Health Organization concerning the legal status of any country, territory, city or area or of its authorities, or concerning the delimitation of its frontiers or boundaries. Dotted lines on maps represent approximate border lines for which there may not yet be full agreement.

The mention of specific companies or of certain manufacturers' products does not imply that they are endorsed or recommended by the World Health Organization in preference to others of a similar nature that are not mentioned. Errors and omissions excepted, the names of proprietary products are distinguished by initial capital letters.

All reasonable precautions have been taken by the World Health Organization to verify the information contained in this publication. However, the published material is being distributed without warranty of any kind, either expressed or implied. The responsibility for the interpretation and use of the material lies with the reader. In no event shall the World Health Organization be liable for damages arising from its use.

Printed in

Contents

Introduction 1

Target audience 2

Settings 2

Resource needs 2

What are the goals of implementing these guidelines ? 2

Who needs referral to a specialist facility? 3

Part 1 **Assessing and managing cardiovascular risk in people with risk factors who have not yet developed clinically manifest cardiovascular disease (primary prevention)** 5

When is grading cardiovascular risk using charts unnecessary for making treatment decisions? 7

Instructions for using WHO/ISH risk prediction charts 7

Practice points 9

Table 1 WHO/ISH risk prediction charts by epidemiological sub-region and WHO Member States 10

Figure 1 WHO/ISH risk prediction chart for AFR D (for use in settings where blood cholesterol can be measured) 12

Figure 2 WHO/ISH risk prediction chart for AFR E (for use in settings where blood cholesterol can be measured) 13

Figure 3 WHO/ISH risk prediction chart for AFR D (for use in settings where blood cholesterol cannot be measured) 14

Figure 4 WHO/ISH risk prediction chart for AFR E (for use in settings where blood cholesterol cannot be measured) 15

Recommendations for prevention of cardiovascular disease in people with risk factors 16

Part 2 **Management of people with established coronary heart disease (CHD), Cerebrovascular disease (CeVD) or peripheral vascular disease (secondary prevention)** 23

Recommendations for prevention of recurrent CHD (heart attack) and CeVD (stroke) events 24

Table 2 Causes and clinical features of secondary hypertension 29

Table 3 Clinical features of malignant hypertension 29

Table 4 Drugs and daily dosages 30

Introduction

Cardiovascular disease (CVD) is a major cause of disability and premature death throughout the world. The underlying pathology is atherosclerosis, which develops over many years and is usually advanced by the time symptoms occur, generally in middle age. Acute coronary events (heart attacks) and cerebrovascular events (strokes) frequently occur suddenly, and are often fatal before medical care can be given. Risk factor modification can reduce clinical events and premature death in people with established cardiovascular disease as well as in those who are at high cardiovascular risk due to one or more risk factors.

These pocket guidelines provide evidence-based guidance on how to reduce the incidence of first and recurrent clinical events due to coronary heart disease (CHD), cerebrovascular disease (CeVD) and peripheral vascular disease in two categories of people. They include;

1. People with risk factors who have not yet developed clinically manifest cardiovascular disease (primary prevention).[1]
2. People with established CHD, CeVD or peripheral vascular disease (secondary prevention).[2]

The accompanying World Health Organization/International Society of Hypertension (WHO/ISH) risk prediction charts enable the estimation of total cardiovascular risk of people in the first category. The evidence-based recommendations given in Part 1 of these guidelines, provide guidance on which specific preventive actions to initiate, and with what degree of intensity.

People in the second category have high cardiovascular risk and need intensive lifestyle interventions and appropriate drug therapy as elaborated in Part II of these guidelines. Risk stratification using risk charts is not required for making treatment decisions in them.

[1] *World Health Organization. Prevention of Cardiovascular Disease. Guidelines for assessment and management of cardiovascular risk. Geneva, 2007.*
[2] *World Health Organization. Prevention of recurrent heart attacks and strokes in low and middle income populations. Evidence-based recommendations for policy makers and health professionals. Geneva, 2003.*

Target audience

These pocket guidelines can be used by physicians and non-physician health workers, at all levels of health care including primary care. Hypertension, diabetes or established cardiovascular disease may be used as entry points for implementing these guidelines.

Settings

Primary care and other levels of care including low resource settings

Resource needs

- *Human resources*: Medical doctor or trained nurse/non-physician health worker
- *Equipment*: Stethoscope, accurate blood pressure measurement device[1], measuring tape and weighing scale, equipment for testing urine glucose and urine albumin, and assay of blood glucose and blood cholesterol
- *Drugs*: Thiazide diuretics, beta blockers, angiotensin converting enzyme inhibitors, calcium channel blockers, aspirin, metformin, insulin, statins
- *Other facilities*: System for maintaining medical records, referral facilities

What are the goals of implementing these guidelines?

The goals are to prevent CHD and CeVD events by lowering cardiovascular risk. The recommendations assist people to:

- quit tobacco use, or reduce the amount smoked, or not start the habit
- make healthy food choices
- be physically active
- reduce body mass index, waist–hip ratio/waist circumference
- lower blood pressure
- lower blood cholesterol and low density lipoprotein cholesterol (LDL-cholesterol)
- control glycaemia
- take antiplatelet therapy when necessary.

[1] Parati G, Mendis S, Abegunde D, Asmar R, Mieke S, Murray A, Shengelia B, Steenvoorden G, Van Montfrans G, O'Brien E; World Health Organization. Recommendations for blood pressure measuring devices for office/clinic use in low resource settings. *Blood Press Monit.* 2005 Feb;10(1):3-10.

Who needs referral to a specialist facility ?

Referral is required if there are clinical features suggestive of:

- acute cardiovascular events such as: heart attack, angina, heart failure, arrhythmias, stroke, transient ischemic attack
- secondary hypertension (table 2), malignant hypertension, (table 3)
- diabetes mellitus (newly diagnosed or uncontrolled)
- established cardiovascular disease (newly diagnosed or if not assessed in a specialist facility).

Once the condition of the above categories of people is assessed and stabilized, they can be followed up in a primary care facility based on the recommendations provided in these pocket guidelines. They will need periodic reassessments in speciality care.

Part 1

Assessing and managing cardiovascular risk in people with risk factors who have not yet developed clinically manifest cardiovascular disease (primary prevention)

When is grading cardiovascular risk using charts unnecessary for making treatment decisions?

Some individuals are at high cardiovascular risk because they have established cardiovascular disease or very high levels of individual risk factors. Risk stratification is not necessary for making treatment decisions for these individuals as they belong to the high risk category; all of them need intensive lifestyle interventions and appropriate drug therapy[1,2]. They include people:

- with established cardiovascular disease
- without established CVD who have a total cholesterol ≥ 8 mmol/l (320 mg/dl) or low-density lipoprotein (LDL) cholesterol ≥ 6 mmol/l (240 mg/dl) or TC/HDL-C (total cholesterol/high density lipoprotein cholesterol) ratio >8
- without established CVD who have persistent raised blood pressure (>160–170/100–105 mmHg)
- with type 1 or 2 diabetes, with overt nephropathy or other significant renal disease
- with renal failure or renal impairment.

Instructions for using WHO/ISH risk prediction charts

The WHO/ISH risk prediction charts indicate 10-year risk of a fatal or non-fatal major cardiovascular event (myocardial infarction or stroke), according to age, sex, blood pressure, smoking status, total blood cholesterol and presence or absence of diabetes mellitus for 14 WHO epidemiological sub-regions.

There are two sets of charts. One set (14 charts) can be used in settings where blood cholesterol can be measured. The other set (14 charts) is for settings in which blood cholesterol cannot be measured. Both sets are available in colour and shades of black on a compact disc.

Each chart can only be used in countries of the specific WHO epidemiological sub-region as shown in table 1.

The charts provide approximate estimates of CVD risk in people who do not have established coronary heart disease, stroke or other atherosclerotic disease. They are useful as tools to help identify those at high cardiovascular risk, and to motivate patients, particularly to change behaviour and, when appropriate, to take antihypertensive, lipid-lowering drugs and aspirin.

---

[1] *World Health Organization. Prevention of recurrent heart attacks and strokes in low and middle income populations. Evidence-based recommendations for policy makers and health professionals. Geneva, 2003.*

[2] *World Health Organization. Avoiding heart attacks and strokes. Don`t be a victim protect yourself. Geneva 2005.*

How do you use the charts to assess cardiovascular risk? (see figures 1, 2, 3, 4)

- First make sure that you select the appropriate charts using information in table 1
- If blood cholesterol cannot be measured due to resource limitations, use the charts that do not have total cholesterol
- Before applying the chart to estimate the 10 year cardiovascular risk of an individual, the following information is necessary
  - Presence or absence of diabetes[1]
  - Gender
  - Smoker or non-smoker[2]
  - Age
  - Systolic blood pressure (SBP)[3]
  - Total blood cholesterol[4] (if in mg/dl divide by 38 to convert to mmol/l).

Once the above information is available proceed to estimate the 10-year cardiovascular risk as follows:

Step 1 Select the appropriate chart depending on the presence or absence of diabetes

Step 2 Select male or female tables

Step 3 Select smoker or non smoker boxes

Step 4 Select age group box (if age is 50-59 years select 50, if 60-69 years select 60 etc)

Step 5 Within this box find the nearest cell where the individuals systolic blood pressure (mm Hg) and total blood cholesterol level (mmol/l) cross. The colour of this cell determines the 10 year cardiovascular risk.

1 A person who has diabetes is defined as someone taking insulin or oral hypoglycaemic drugs, or with a fasting plasma glucose concentration above 7.0 mmol/l (126 mg/dl) or a postprandial (approximately 2 hours after a main meal) plasma glucose concentration above 11.0 mmol/l (200 mg/l)on two separate occasions). For very low resource settings urine sugar test may be used to screen for diabetes if blood glucose assay is not feasible. If urine sugar test is positive a confirmatory blood glucose test need to be arranged to diagnose diabetes mellitus.

2 All current smokers and those who quit smoking less than 1 year before the assessment are considered smokers for assessing cardiovascular risk.

3 Systolic blood pressure, taken as the mean of two readings on each of two occasions, is sufficient for assessing risk but not for establishing a pretreatment baseline.

4 The mean of two non-fasting measurements of serum cholesterol by dry chemistry, or one non-fasting laboratory measurement, is sufficient for assessing risk.

Practice points

Please note that CVD risk may be higher than indicated by the charts in the presence of the following:

- already on antihypertensive therapy
- premature menopause
- approaching the next age category or systolic blood pressure category
- obesity (including central obesity)
- sedentary lifestyle
- family history of premature CHD or stroke in first degree relative (male < 55 years, female < 65 years)
- raised triglyceride level (>2.0 mmol/l or 180 mg/dl)
- low HDL cholesterol level (< 1 mmol/l or 40mg/dl in males, < 1.3 mmol/l or 50 mg/dl in females)
- raised levels of C-reactive protein, fibrinogen, homocysteine, apolipoprotein B or Lp(a), or fasting glycaemia, or impaired glucose tolerance
- microalbuminuria (increases the 5-year risk of diabetics by about 5%)
- raised pulse rate
- socioeconomic deprivation.

Table 1. List of WHO/ISH risk prediction charts by epidemiological sub-regions[1] and WHO Member States

WHO/ISH risk prediction charts by epidemiological sub regions		WHO Member States
**Africa**	**AFR D**	Algeria, Angola, Benin, Burkina Faso, Cameroon, Cape Verde, Chad, Comoros, Equatorial Guinea, Gabon, Gambia, Ghana, Guinea, Guinea-Bissau, Liberia, Madagascar, Mali, Mauritania, Mauritius, Niger, Nigeria, Sao Tome And Principe, Senegal, Seychelles, Sierra Leone, Togo
	**AFR E**	Botswana, Burundi, Central African Republic, Congo, Côte d'Ivoire, Democratic Republic of The Congo, Eritrea, Ethiopia, Kenya, Lesotho, Malawi, Mozambique, Namibia, Rwanda, South Africa, Swaziland, Uganda, United Republic of Tanzania, Zambia, Zimbabwe
**The Americas**	**AMR A**	Canada*, Cuba, United States of America*,
	**AMR B**	Antigua and Barbuda, Argentina, Bahamas, Barbados, Belize, Brazil, Chile, Colombia, Costa Rica, Dominica, Dominican Republic, El Salvador, Grenada, Guyana, Honduras, Jamaica, Mexico, Panama, Paraguay, Saint Kitts And Nevis, Saint Lucia, Saint Vincent and the Grenadines, Suriname, Trinidad and Tobago, Uruguay, Venezuela
	**AMR D**	Bolivia, Ecuador, Guatemala, Haiti, Nicaragua, Peru
**Eastern Mediterranean**	**EMR B**	Bahrain, Iran (Islamic Republic of), Jordan, Kuwait, Lebanon, Libyan Arab Jamahiriya, Oman, Qatar, Saudi Arabia, Syrian Arab Republic, Tunisia, United Arab Emirates
	**EMR D**	Afghanistan, Djibouti, Egypt, Iraq, Morocco, Pakistan, Somalia, Sudan, Yemen

[1] Mortality strata: A: very low child mortality and very low adult mortality; B: low child mortality and low adult mortality; C: low child mortality and high adult mortality; D: high child mortality and high adult mortality; E: high child mortality and very high adult mortality.

* Other risk prediction charts are already available for Australia, Canada, New Zealand , the United States of America and many countries in Europe.

WHO/ISH risk prediction charts by epidemiological sub regions		WHO Member States
**Europe***	**EUR A**	Andorra, Austria, Belgium, Croatia, Cyprus, Czech Republic, Denmark, Finland, France, Germany, Greece, Iceland, Ireland, Israel, Italy, Luxembourg, Malta, Monaco, Netherlands, Norway, Portugal, San Marino, Slovenia, Spain, Sweden, Switzerland, United Kingdom
	**EUR B**	Albania, Armenia, Azerbaijan, Bosnia And Herzegovina, Bulgaria, Georgia, Kyrgyzstan, Poland, Romania, Serbia and Montenegro, Slovakia, Tajikistan, The Former Yugoslav Republic of Macedonia, Turkey, Turkmenistan, Uzbekistan
	**EUR C**	Belarus, Estonia, Hungary, Kazakhstan, Latvia, Lithuania, Republic of Moldova, Russian Federation, Ukraine
**South-East Asia**	**SEAR B**	Indonesia, Sri Lanka, Thailand
	**SEAR D**	Bangladesh, Bhutan, Democratic People's Republic of Korea, India, Maldives, Myanmar, Nepal
**Western Pacific**	**WPR A**	Australia*, Brunei Darussalam, Japan, New Zealand*, Singapore
	**WPR B**	Cambodia, China, Cook Islands, Democratic People's Republic of Korea, Fiji, Kiribati, Lao People's Democratic Republic, Malaysia, Marshall Islands, Micronesia (Federated States of), Mongolia, Nauru, Niue, Palau, Papua New Guinea, Philippines, Samoa, Solomon Islands, Tonga, Tuvalu, Vanuatu, Viet Nam

Figure 1. WHO/ISH risk prediction chart for AFR D. 10-year risk of a fatal or non-fatal cardiovascular event by gender, age, systolic blood pressure, total blood cholesterol, smoking status and presence or absence of diabetes mellitus.

Risk Level <10% | 10% to <20% | 20% to <30% | 30% to <40% | ≥40%

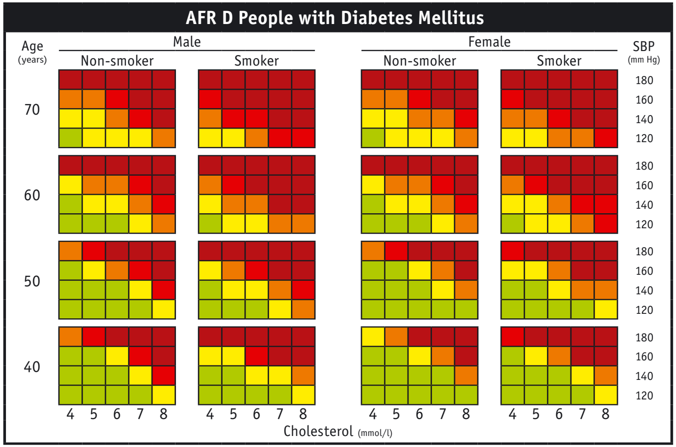

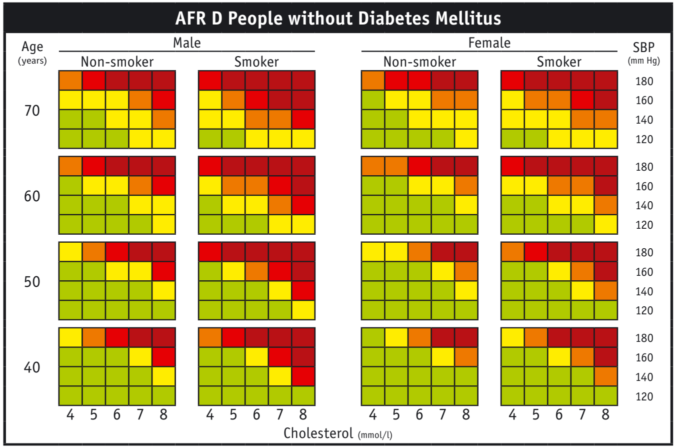

This chart can only be used for countries of the WHO Region of Africa, sub-region D, in settings where blood cholesterol can be measured (see Table 1).

Figure 2. WHO/ISH risk prediction chart for AFR E. 10-year risk of a fatal or non-fatal cardiovascular event by gender, age, systolic blood pressure, total blood cholesterol, smoking status and presence or absence of diabetes mellitus.

Risk Level <10% | 10% to <20% | 20% to <30% | 30% to <40% | ≥40%

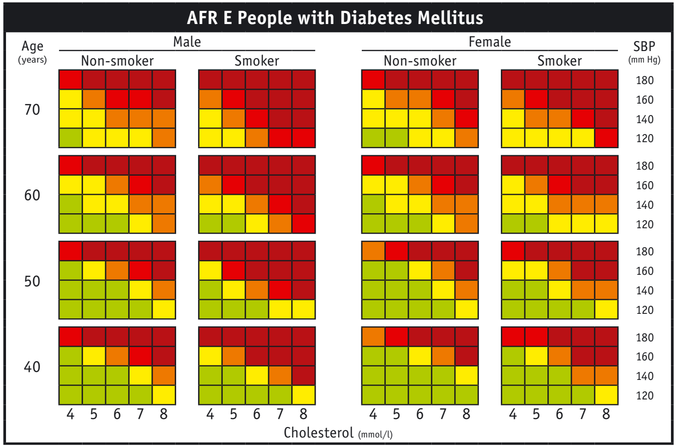

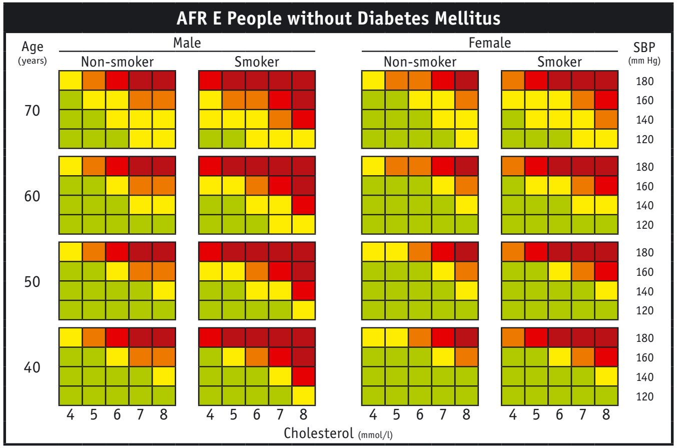

This chart can only be used for countries of the WHO Region of Africa, sub-region E, in settings where blood cholesterol can be measured (see Table 1).

Figure 3. WHO/ISH risk prediction chart for AFR D. 10-year risk of a fatal or non-fatal cardiovascular event by gender, age, systolic blood pressure, smoking status and presence or absence of diabetes mellitus.

Risk Level <10% | 10% to <20% | 20% to <30% | 30% to <40% | ≥40%

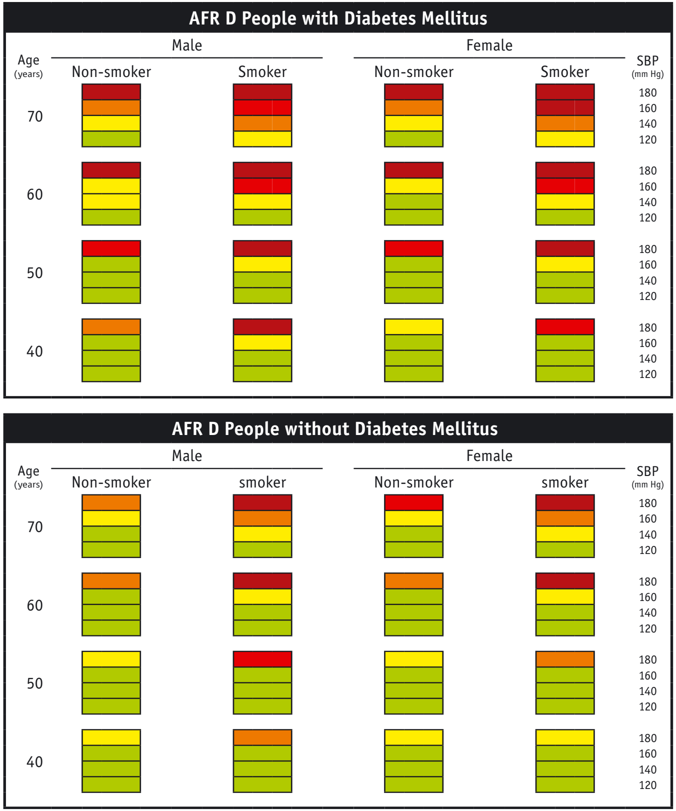

This chart can only be used for countries of the WHO Region of Africa, sub-region D, in settings where blood cholesterol CANNOT be measured (see Table 1).

Figure 4. WHO/ISH risk prediction chart for AFR E. 10-year risk of a fatal or non-fatal cardiovascular event by gender, age, systolic blood pressure, smoking status and presence or absence of diabetes mellitus.

Risk Level <10% | 10% to <20% | 20% to <30% | 30% to <40% | ≥40%

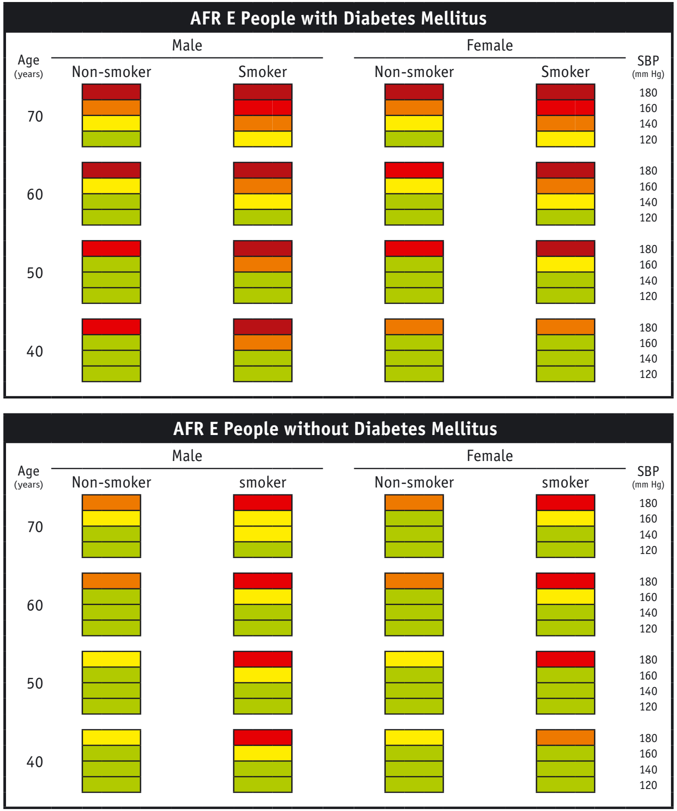

Note: This chart can only be used for countries of the WHO Region of South-East Asia, sub-region D, in settings where blood cholesterol CANNOT be measured. (see Table 1)

Recommendations* for prevention of cardiovascular disease in people with cardiovascular risk factors (according to individual total risk)[a]

**10 year risk of cardiovascular event**<br>**<10%, 10 to <20%, 20 to <30%, ≥30%**	
When resources are limited, individual counselling and provision of care may have to be prioritized according to cardiovascular risk.	
**Risk <10%**	Individuals in this category are at low risk. Low risk does not mean "no" risk.<br>Conservative management focusing on lifestyle interventions is suggested[b].
**Risk 10% to <20%**	Individuals in this category are at moderate risk of fatal or non-fatal vascular events.<br>Monitor risk profile every 6–12 months.
**Risk 20% to <30%**	Individuals in this category are at high risk of fatal or non-fatal vascular events.<br>Monitor risk profile every 3–6 months.
**Risk ≥30%**	Individuals in this category are at very high risk of fatal or non-fatal vascular events.<br>Monitor risk profile every 3–6 months

* For levels of evidence and grades of recommendations see reference 1.
a Excluding people with established CHD, CeVD and peripheral vascular disease
b Policy measures that create conducive environments for quitting tobacco, engaging in physical activity and consuming healthy diets are necessary to promote behavioural change. They will benefit the whole population. For individuals in low risk categories, they can have a health impact at lower cost, compared to individual counselling and therapeutic approaches.

SMOKING CESSATION	
All nonsmokers should be encouraged not to start smoking.<br>All smokers should be strongly encouraged to quit smoking by a health professional and supported in their efforts to do so. (1++, A)<br>It is suggested that those who use other forms of tobacco be advised to stop. (2+, C)	
**Risk 20% to <30%**	Nicotine replacement therapy and/or nortriptyline or amfebutamone (bupropion) should be offered to motivated smokers who fail to quit with counselling. (1++, B)
**Risk ≥30%**	Nicotine replacement therapy and/or nortriptyline or amfebutamone (bupropion) should be offered to motivated smokers who fail to quit with counselling. (1++, B)

DIETARY CHANGES
All individuals should be strongly encouraged to reduce total fat and saturated fat intake. (1+, A)
Total fat intake should be reduced to about 30% of calories, saturated fat to less than 10% of calories, transfatty acids intake should be reduced as much as possible or eliminated and most dietary fat should be polyunsaturated (up to 10% of calories) or monounsaturated (10–15% of calories). (1+, A)
All individuals should be strongly encouraged to reduce daily salt intake by at least one third and, if possible, to <5 g or <90 mmol per day. (1+, A)
All individuals should be encouraged to eat at least 400 g a day of a range of fruits and vegetables as well as whole grains and pulses. (2+, A)
**PHYSICAL ACTIVITY**
All individuals should be strongly encouraged to take at least 30 minutes of moderate physical activity (e.g. brisk walking) a day, through leisure time, daily tasks and work-related physical activity. (1+, A)
**WEIGHT CONTROL**
All individuals who are overweight or obese should be encouraged to lose weight through a combination of a reduced-energy diet (dietary advice) and increased physical activity. (1+, A)
**ALCOHOL INTAKE**
Individuals who take more than 3 units of alcohol[c] per day should be advised to reduce alcohol consumption. (2++, B)

[c] One unit (drink) = half pint of beer/lager (5 % alcohol), 100 ml of wine (10 % alcohol), spirits 25 ml (40% alcohol)

ANTIHYPERTENSIVE DRUGS[√]	
All individuals with blood pressure at or above 160/100 mmHg, or lesser degree of raised blood pressure with target organ damage, should have drug treatment and specific lifestyle advice to lower their blood pressure and risk of cardiovascular disease. (2++, B)	
All individuals with blood pressure below 160/100 mmHg, or with no target organ damage need to be managed according to the cardiovascular risk (10 year risk of cardiovascular event <10%, 10 to <20%, 20 to <30%, ≥30%)	
**Risk <10%**	Individuals with persistent blood pressure ≥140/90 mmHg[e] should continue lifestyle strategies to lower blood pressure and have their blood pressure and total cardiovascular risk reassessed every 2–5 years depending on clinical circumstances and resource availability.
**Risk 10% to <20%**	Individuals with persistent blood pressure ≥140/90 mmHg[e] should continue lifestyle strategies to lower blood pressure and have their blood pressure and total cardiovascular risk reassessed annually depending on clinical circumstances and resource availability.
**Risk 20% to <30%**	Individuals with persistent blood pressure ≥140/90 mmHg[e] who are unable to lower blood pressure through lifestyle strategies with professional assistance within 4–6 months should be considered for one of the following drugs[√] to reduce blood pressure and risk of cardiovascular disease: thiazide-like diuretic, ACE inhibitor, calcium channel blocker, beta-blocker[d].<br>A low-dose thiazide-like diuretic, ACE inhibitor or calcium channel blocker is recommended as first-line therapy. (1++, A)

ANTIHYPERTENSIVE DRUGS√	
**Risk ≥30%**	Individuals with persistent blood pressure ≥130/80 mmHg should be given one of the following drugs to reduce blood pressure and risk of cardiovascular disease: thiazide-like diuretic, ACE inhibitor, calcium channel blocker, beta-blocker[d].<br>A low-dose thiazide-like diuretic, ACE inhibitor or calcium channel blocker is recommended as first-line therapy. (1++, A)

[d] Evidence from two recent meta-analyses indicates that for treatment of hypertension, beta-blockers are inferior to calcium-channel blockers and ACE inhibitors in reducing the frequency of hard endpoints. In addition, beta-blockers are less well tolerated than diuretics. Most of this evidence comes from trials where atenolol was the beta-blocker used.

[e] Reducing blood pressure by 10–15/5–8 mmHg with drug treatment reduces combined CVD mortality and morbidity by about one-third, whatever the pretreatment absolute risk. However, applying this recommendation will lead to a large proportion of the adult population receiving antihypertensive drugs. Even in some high-resource settings, current practice is to recommend drugs for this group only if the blood pressure is at or above 160/100 mmHg.

LIPID-LOWERING DRUGS (STATINS)√	
All individuals with total cholesterol at or above 8 mmol/l (320 mg/dl) should be advised to follow a lipid-lowering diet and given a statin to lower the risk of cardiovascular disease. (2++, B)	
All other individuals need to be managed according to the cardiovascular risk as follows<br>(10 year risk of cardiovascular event <10%, 10 to <20%, 20 to 30%, ≥30%)	
**Risk <10%**	Should be advised to follow a lipid-lowering diet[g].
**Risk 10 to <20%**	Should be advised to follow a lipid-lowering diet[g].
**Risk 20 to <30%**	Adults >40 years with persistently high serum cholesterol (>5.0 mmol/l) and/or LDL cholesterol >3.0 mmol/l, despite a lipid-lowering diet, should be given a statin. (1+, A)
**Risk ≥30%**	Individuals in this risk category should be advised to follow a lipid-lowering diet and given a statin. (1++, A)<br>Serum cholesterol should be reduced to less than 5.0 mmol/l (LDL cholesterol to below 3.0 mmol/l) or by 25% (30% for LDL cholesterol), whichever is greater[f].
**HYPOGLYCAEMIC DRUGS√**	
Individuals with persistent fasting blood glucose >6 mmol/l despite diet control should be given metformin. (1+, A)	

[f] Reducing cholesterol level by 20% (approximately 1 mmol/l) with statin treatment would be expected to yield a coronary heart disease mortality benefit of 30%, whatever the pretreatment absolute risk. However, applying this to the general population may not be cost effective. It will lead to a large proportion of the adult population receiving statins. Even in some high-resource settings, current practice is to recommend drugs for this group only if serum cholesterol is above 8mmol/l (320 mg/dl).

[g] There are no clinical trials that have evaluated the absolute and relative benefits of cholesterol lowering to different cholesterol targets in relation to clinical events.

ANTIPLATELET DRUGS[√]	
**Risk <10%**	For individuals in this risk category, the harm caused by aspirin treatment outweighs the benefits.<br>Aspirin should *not* be given to individuals in this low-risk category. (1++, A)
**Risk 10 to <20%**	For individuals in this risk category, the benefits of aspirin treatment are balanced by the harm caused.<br>Aspirin should *not* be given to individuals in this risk category. (1++, A)
**Risk 20 to <30%**	For individuals in this risk category, the balance of benefits and harm from aspirin treatment is not clear[h].<br>Aspirin should probably *not* be given to individuals in this risk category. (1++, A)
**Risk ≥30%**	Individuals in this risk category should be given low-dose aspirin. (1++, A)
DRUGS THAT ARE NOT RECOMMENDED	
	Hormone replacement, vitamins B, C, E and folic acid supplements are not recommended for reduction of cardiovascular risk.

[h] Consider aspirin in areas where coronary heart disease rates exceed stroke rates.
[√] Best Practice points: Unless there are compelling indications to use specific drugs, the least expensive preparation of the above classes of drugs should be used. Good quality generic preparations of medicines listed in WHO essential medicines list are recommended.

The most cost effective preventive treatments are aspirin and initial antihypertensive treatment (low dose thiazides). Intensive antihypertensive treatment and stain treatment are less cost effective. In limited resource settings a cost effective prevention strategy could offer aspirin and initial antihypertensive treatment to all at high risk before offering intensive antihypertensive treatment and statins.

Part 2

Management of people with established CHD, CeVD or peripheral vascular disease (secondary prevention)

People with established cardiovascular disease (angina pectoris, coronary heart disease, myocardial infarction, transient ischaemic attacks, cerebrovascular disease (CeVD) or peripheral vascular disease (PVD) or after coronary revascularization or carotid endarterectomy) are at very high risk of developing recurrent cardiovascular events. Risk charts are not necessary to make treatment decisions in them.

The goal of applying the recommendations below, is to prevent recurrent cardiovascular events by reducing their cardiovascular risk.

Recommendations* for prevention of recurrent CHD (heart attacks) and CeVD (strokes) events

LIFESTYLE ADVICE
Intensive life style advice should be given simultaneously with drug treatment
**SMOKING CESSATION**
All individuals with established CHD and/or CeVD should be strongly encouraged to stop smoking by a health professional and supported in their efforts to do so. (IIa B)<br>Cessation of other forms of tobacco use in individuals with established CHD and /or CeVD is recommended. (IIa C)<br>Nicotine replacement therapy should be offered to individuals who continue to smoke at least 10 cigarettes a day or more, who are likely to be markedly nicotine dependent. The use of antidepressants for smoking cessation is not generally recommended for patients with CHD and /or CeVD (Ia B)<br>Non-smoking people with CHD and/or CeVD should be advised to avoid exposure to second-hand tobacco smoke as much as possible. (IIa C)

DIETARY CHANGES
All individuals with CHD and/or CeVD should be given advice to adopt a pattern of diet which is likely to reduce the risk of recurrent vascular disease.<br>Total fat intake should be reduced to < 30% of calories, saturated fat to < 10% of calories and transfatty acids should be reduced as much as possible or eliminated; most dietary fat should be polyunsaturated (up to 10% of calories) or monounsaturated (10–15% of calories). (IIa B)<br>All individuals should be strongly encouraged to reduce daily salt intake by at least one-third and, if possible, to <5 g or <90 mmol per day. (IIa B)<br>All individuals should be encouraged to eat, at least 400 g a day, of a range fruits and vegetables, as well as whole grains and pulses. (1a, A)
**PHYSICAL ACTIVITY**
Regular light to moderate intensity physical exercise is recommended for all subjects recovering from major CHD events (including coronary revascularization). (Ia A)<br>Supervised programmes of exercise should where feasible be offered to all subjects recovering from major CHD events and CeVD events. (Ia A)
**WEIGHT CONTROL**
In patients with cardiovascular disease who are overweight or obese, weight loss should be advised through the combination of a reduced energy diet and increased physical activity. (IIa B)
**ALCOHOL INTAKE**
Individuals who take more than 3 units of alcohol[c] per day should be advised to reduce alcohol consumption. (2++, B)

[c] One unit (drink) = half pint of beer/lager (5 % alcohol), 100 ml of wine (10 % alcohol), spirits 25 ml (40% alcohol)

ANTIHYPERTENSIVE DRUGS√

Blood pressure reduction should be considered in all patients with established CHD, particularly with a blood pressure level above 140/90 mmHg. Lifestyle factors (particularly high alcohol intake) should be addressed first and if blood pressure is still above 140/90 mmHg, drug treatment is indicated. When beta-blockers and ACEI (angiotensin converting enzyme inhibitors) cannot be given, or in cases where blood pressure remains high, treatment with a thiazide diuretic is likely to reduce risk of recurrent vascular events. A target blood pressure of 130/80-85 mmHg is appropriate.

Blood pressure reduction should be considered in all patients with previous TIA or stroke to a target of <130/<80-85 mmHg (Ia A).

LIPID LOWERING DRUGS√

Treatment with statins is recommended for all patients with established CHD. Treatment should be continued in the long term, probably lifelong. Patients at high baseline risk are particularly likely to benefit. (Ia A)

Treatment with a statin should be considered for all patients with established CeVD, especially if they also have evidence of established CHD. (Ia A)

Monitoring of blood cholesterol levels is not mandatory. A total cholesterol of less than 4.0 mmol/l (152 mg/dl) and LDL-cholesterol of less than 2.0 mmol/l (77 mg/dl), or a reduction of 25% in total cholesterol and 30% in LDL-cholesterol, whichever achieves the lower absolute risk level, may be desirable goals.

Other lipid lowering agents are not recommended, either as an alternative to statins or in addition to them. (Ia A)

HYPOGLYCEMIC DRUGS√

Secondary prevention of CHD, CeVD and PVD is important in patients with diabetes, whether type 1 or type 2. Individuals with persistent fasting blood glucose >6 mmol/l despite diet control should be given metformin and/or insulin as appropriate. (1+, A).

ANTIPLATELET DRUGS√

All patients with established CHD should be treated with regular aspirin in the absence of clear contraindications. Treatment should be initiated early and continued lifelong. (Ia A)

All patients with a history of transient ischaemic attack or stroke presumed due to cerebral ischaemia or infarction should be treated with long-term (probably lifelong) aspirin in the absence of clear contraindications. (Ia A)

FOLLOWING MYOCARDIAL INFARCTION AN ACEI√

ACE inhibitors are recommended in all patients following myocardial infarction, which should be initiated as early as possible and continued long-term, probably lifelong. The benefits of treatment are particularly great among patients with impaired left ventricular function. (Ia A)

FOLLOWING MYOCARDIAL INFARCTION A BETA BLOCKER√

Treatment with beta-blockers is recommended in all patients with a history of myocardial infarction and those with CHD who have developed major left ventricular dysfunction leading to heart failure. (Ia A) Treatment should be continued for a minimum of 1–2 years after MI and probably lifelong, unless serious side effects occur. Beta-blockers are probably beneficial in patients with angina, although robust data are lacking.

ANTICOAGULANT TREATMENT√

Long term anticoagulation is not recommended in patients with a history of stroke or TIA who are in sinus rhythm. (Ia A)

Long term anticoagulation is recommended for patients with a history of stroke or TIA who are in atrial fibrillation, at low risk of bleeding and in whom treatment with anticoagulants can be safely monitored. In circumstances in which anticoagulant monitoring is not possible, or if a patient cannot take anticoagulants, treatment with aspirin should be offered. (Ia A)

CORONARY REVASCULARIZATION
CABG (coronary artery bypass graft) surgery should be considered as an adjunct to optimal medical treatment including aspirin, lipid lowering treatment, ACE inhibitors and beta-blockers) in those patients at moderate and high risk who are considered likely to have left main stem or triple vessel disease. (Ia A)<br>PTCA (percutaneous coronary angioplasty) should be considered for relief of anginal symptoms in patients with refractory angina who are already receiving optimal medical treatment. (Ia A)
**CAROTID ENDARTERECTOMY**
Carotid endarterectomy reduces the risk of recurrent stroke and death among patients with a previous TIA or non-disabling stroke in patients with severe ipsilateral carotid stenosis (70–99%) and possibly in patients with moderate degrees of stenosis (50–69%) though not in milder degrees of stenosis. (Ia A)
**DRUGS THAT ARE NOT RECOMMENDED**
On the basis of current evidence, treatment with type 1 anti-arrhythmics, calcium channel blockers, antioxidant vitamins, folate and hormone replacement therapy are not recommended for CHD or CeVD patients.

* For levels of evidence and grades of recommendations see reference 2.

√ Best Practice point: Unless there are compelling indications to use specific drugs, the least expensive preparation of the above classes of drugs should be used. Good quality generic preparations of medicines listed in WHO essential medicines list are recommended

Table 2 Causes and clinical features of secondary hypertension

Cause	Clinical features
**Kidney diseases:**	History of episodes of blood or proteins in the urine, urinary infections, swelling of body
**Nephropathy**	Kidney disease in the family (polycystic kidney disease)
**Renal artery stenosis**	Physical examination: abdomen or loin bruit, palpable kidneys
**Phaeochromocytoma**	Episodic symptoms: headache, flushing, sweating
**Cushing syndrome**	High blood pressure fluctuant and sudden onset
**Conn syndrome**	Typical general appearance: truncal obesity, stretch marks,
**Acromegaly**	Weakness, cramps, polyuria
	Tall stature, typical facies with prominent lower jaw, broad spade shaped hands
**Coarctation of the aorta**	High blood pressure in upper limbs but not in lower limbs. Delayed or weak femoral pulses
**Drugs**	Contraceptive pill, anti-inflammatory drugs, steroids, sympathomimetics, nasal decongestants, appetite suppressants, cyclosporine, erythropoietin, liquorice, antidepressants

Table 3 Clinical features of malignant hypertension

**Symptoms**	Headache, blurred vision, nausea, vomiting, lethargy, chest-pain, dyspnoea, seizure, mental status changes, oliguria
**Physical signs**	Abrupt rise in blood pressure (diastolic blood pressure often > 120mmHg)<br>Retinal examination: papilloedema, flame-shaped retinal haemorrhages, soft exudates,<br>Signs of congestive heart failure and pulmonary oedema<br>Neurological deficits

Table 4 Drugs and daily dosages

Class of drug	Drug	Daily dosage
ACEI<br>(Angiotensin converting enzyme inhibitors)	Captopril<br>Enalapril	*initial dose 6.25 to 12.5 mg three times daily, increasing to 25–50 mg three times daily*<br>*initial dose 2.5–5.0 mg twice daily, increasing to 10–20 mg twice daily*
CCBs (Calcium Channel Blockers)	Nifedipine<br>(sustained release formulations)	*Starting at 30 mg increasing to 120 mg once daily*
Thiazide diuretics	Hypdrocholorothiazide<br>Bendrofluazide	*Starting at 12.5 mg increasing to 25 mg once daily*<br>*2.5 mg as a single daily dose.*
Beta-blockers	Propranolol<br>Atenolol<br>Metoprolol	*80 mg twice daily*<br>*Starting at 50 mg to 100mg once daily*<br>50–100 mg twice daily
Lipid lowering therapy	Simvastatin,	*Initial dose 10 mg once at night, increasing to 40 mg once at night*
Antiplatelet therapy	Aspirin	*Starting at 75-100 mg daily*
Hypoglycemic drugs	Glibenclamide<br>Metformin	*Starting at 2.5 mg increasing to 5 mg twice daily before meals*<br>*Starting at 0.5 g increasing to 1.0 g three times daily*

CVD
CARDIOVASCULAR DISEASES

WHO/ISH risk prediction charts for all other epidemiological sub-regions are available on compact disc and at the WHO website:

http://www.who.int/cardiovascular_diseases

The material in these pocket guidelines was adapted from WHO guidelines on primary and secondary prevention available on WHO website:

World Health Organization. Prevention of Cardiovascular Disease. Guidelines for assessment and management of cardiovascular risk. Geneva, 2007.

World Health Organization. Prevention of recurrent heart attacks and strokes in low- and middle-income populations. Evidence-based recommendations for policy-makers and health professionals. Geneva, 2003.

ISBN 978 92 4 154726 0

Contact information for training modules

Tel: +41 22 791 3441

E mail: mendiss @who.int

9 789241 547260
