# Supplementary material for: Managing hypertension in rural Uganda: Realities and strategies 10 years of experience at a district hospital chronic disease clinic
Source: PLoS One. 2020 Jun 5;15(6):e0234049. doi: 10.1371/journal.pone.0234049 (PMC7274420; doi:10.1371/journal.pone.0234049)
Supplement: S2 File — (PDF) [file pone.0234049.s004.pdf]

WHO cardiovascular disease risk laboratory-based charts

Eastern Sub-Saharan Africa

Burundi, Comoros, Djibouti, Eritrea, Ethiopia, Kenya, Madagascar, Mozambique, Malawi, Rwanda, Somalia, Tanzania, Uganda, Zambia

Risk Level: <5% | 5% to <10% | 10% to <20% | 20% to <30% | ≥30%

People without Diabetes

Age (years)	SBP (mmHg)	Men Non-smoker <4	4-4.9	5-5.9	6-6.9	≥ 7	Men Smoker <4	4-4.9	5-5.9	6-6.9	≥ 7	Women Non-smoker <4	4-4.9	5-5.9	6-6.9	≥ 7	Women Smoker <4	4-4.9	5-5.9	6-6.9	≥ 7
70-74	≥180	22	24	27	29	32	28	30	33	36	39	16	17	17	18	19	22	23	24	25	26
70-74	160-179	18	20	22	24	26	23	25	28	30	33	14	14	15	15	16	19	20	20	21	22
70-74	140-159	15	17	18	20	22	19	21	23	25	28	12	12	13	13	14	16	17	17	18	19
70-74	120-139	12	14	15	16	18	16	17	19	21	23	10	10	11	11	12	14	14	15	15	16
70-74	<120	10	11	12	14	15	13	14	16	17	19	8	9	9	9	10	12	12	12	13	14
65-69	≥180	18	19	21	23	26	23	26	28	31	34	12	13	14	14	15	19	19	20	21	23
65-69	160-179	14	16	17	19	21	19	21	23	25	28	10	11	11	12	13	15	16	17	18	19
65-69	140-159	11	12	14	15	17	15	17	19	21	23	9	9	9	10	10	13	13	14	15	16
65-69	120-139	9	10	11	12	14	12	14	15	17	19	7	7	8	8	9	11	11	12	12	13
65-69	<120	7	8	9	10	11	10	11	12	14	15	6	6	6	7	7	9	9	10	10	11
60-64	≥180	14	15	17	19	21	19	21	24	27	30	10	10	11	11	12	15	16	17	18	20
60-64	160-179	11	12	13	15	17	15	17	19	21	24	8	8	9	9	10	13	13	14	15	16
60-64	140-159	8	9	10	12	13	12	13	15	17	19	6	7	7	8	8	10	11	12	12	13
60-64	120-139	7	7	8	9	10	9	11	12	14	15	5	5	6	6	7	8	9	9	10	11
60-64	<120	5	6	6	7	8	7	8	9	11	12	4	4	5	5	5	7	7	8	8	9
55-59	≥180	11	12	13	15	17	16	18	20	23	26	7	8	8	9	10	13	14	15	16	17
55-59	160-179	8	9	10	11	13	12	14	16	18	20	6	6	7	7	8	10	11	12	13	14
55-59	140-159	6	7	8	9	10	10	11	12	14	16	5	5	5	6	6	8	9	9	10	11
55-59	120-139	5	5	6	7	8	7	8	9	11	13	4	4	4	5	5	6	7	8	8	9
55-59	<120	4	4	5	5	6	6	6	7	8	10	3	3	3	4	4	5	6	6	7	7
50-54	≥180	8	9	10	12	13	13	15	17	19	22	6	6	7	7	8	11	12	12	14	15
50-54	160-179	6	7	8	9	10	10	11	13	15	17	4	5	5	6	6	8	9	10	11	12
50-54	140-159	5	5	6	7	8	8	9	10	11	13	3	4	4	4	5	6	7	8	8	9
50-54	120-139	3	4	4	5	6	6	6	7	9	10	3	3	3	3	4	5	5	6	7	7
50-54	<120	3	3	3	4	5	4	5	6	7	8	2	2	2	3	3	4	4	5	5	6
45-49	≥180	6	7	8	9	11	11	12	14	16	19	4	5	5	6	6	9	10	11	12	13
45-49	160-179	5	5	6	7	8	8	9	11	12	14	3	4	4	4	5	7	7	8	9	10
45-49	140-159	3	4	4	5	6	6	7	8	9	11	2	3	3	3	4	5	6	6	7	8
45-49	120-139	2	3	3	4	4	4	5	6	7	8	2	2	2	2	3	4	4	5	5	6
45-49	<120	2	2	2	3	3	3	4	4	5	6	1	2	2	2	2	3	3	4	4	5
40-44	≥180	5	6	6	7	9	9	10	12	14	16	3	4	4	4	5	7	8	9	10	11
40-44	160-179	3	4	4	5	6	6	7	9	10	12	2	3	3	3	4	5	6	7	8	8
40-44	140-159	2	3	3	4	5	5	5	6	7	9	2	2	2	2	3	4	5	5	6	6
40-44	120-139	2	2	2	3	3	3	4	5	5	7	1	1	2	2	2	3	3	4	4	5
40-44	<120	1	1	2	2	2	2	3	3	4	5	1	1	1	1	2	2	3	3	3	4

Total cholesterol (mmol/l)

People with Diabetes

Age (years)	SBP (mmHg)	Men Non-smoker <4	4-4.9	5-5.9	6-6.9	≥ 7	Men Smoker <4	4-4.9	5-5.9	6-6.9	≥ 7	Women Non-smoker <4	4-4.9	5-5.9	6-6.9	≥ 7	Women Smoker <4	4-4.9	5-5.9	6-6.9	≥ 7
70-74	≥180	30	32	35	38	42	37	40	43	47	51	24	25	25	26	28	32	33	34	36	37
70-74	160-179	25	27	29	32	35	31	34	37	40	43	20	21	22	23	24	27	28	30	31	32
70-74	140-159	20	22	25	27	30	26	28	31	34	37	17	18	19	19	20	24	24	25	26	28
70-74	120-139	17	19	20	23	25	21	23	26	28	31	15	15	16	16	17	20	21	22	23	24
70-74	<120	14	15	17	19	21	18	19	22	24	26	12	13	13	14	15	17	18	19	19	20
65-69	≥180	25	27	30	33	36	33	36	39	43	47	20	21	22	23	24	29	30	31	33	34
65-69	160-179	20	22	25	27	30	27	30	33	36	40	16	17	18	19	20	24	25	27	28	29
65-69	140-159	16	18	20	22	25	22	24	27	30	33	14	14	15	16	17	20	21	22	24	25
65-69	120-139	13	15	16	18	20	18	20	22	25	27	11	12	13	13	14	17	18	19	20	21
65-69	<120	11	12	13	15	17	14	16	18	20	23	9	10	10	11	12	14	15	16	17	18
60-64	≥180	21	23	25	28	32	29	32	35	39	44	16	17	18	19	20	26	27	29	30	32
60-64	160-179	16	18	20	23	26	23	26	29	32	36	13	14	15	16	17	21	22	24	25	27
60-64	140-159	13	14	16	18	21	19	21	23	26	30	11	11	12	13	14	17	18	20	21	22
60-64	120-139	10	11	13	15	17	15	17	19	21	24	9	9	10	11	11	14	15	16	17	19
60-64	<120	8	9	10	12	13	12	13	15	17	19	7	8	8	9	9	12	12	13	14	15
55-59	≥180	17	19	22	24	27	26	29	32	36	40	13	14	15	16	18	23	24	26	28	30
55-59	160-179	13	15	17	19	22	20	23	26	29	33	11	11	12	13	14	18	20	21	23	25
55-59	140-159	10	12	13	15	17	16	18	20	23	26	8	9	10	11	11	15	16	17	19	20
55-59	120-139	8	9	10	12	13	12	14	16	18	21	7	7	8	8	9	12	13	14	15	16
55-59	<120	6	7	8	9	10	9	11	12	14	17	5	6	6	7	7	10	10	11	12	13
50-54	≥180	15	16	18	21	23	23	26	29	33	37	11	12	13	14	15	20	22	24	26	28
50-54	160-179	11	12	14	16	18	18	20	23	26	30	9	9	10	11	12	16	17	19	21	23
50-54	140-159	8	9	11	12	14	13	15	17	20	23	7	7	8	9	9	13	14	15	16	18
50-54	120-139	6	7	8	9	11	10	12	13	16	18	5	6	6	7	7	10	11	12	13	14
50-54	<120	5	5	6	7	8	8	9	10	12	14	4	4	5	5	6	8	9	9	10	12
45-49	≥180	12	14	15	17	20	21	23	26	30	34	9	10	11	12	13	18	20	22	24	26
45-49	160-179	9	10	11	13	15	15	17	20	23	27	7	7	8	9	10	14	15	17	19	21
45-49	140-159	6	7	8	10	12	11	13	15	18	21	5	6	6	7	8	11	12	13	15	16
45-49	120-139	5	5	6	7	9	8	10	11	13	16	4	4	5	5	6	8	9	10	11	13
45-49	<120	3	4	5	5	7	6	7	8	10	12	3	3	4	4	5	6	7	8	9	10
40-44	≥180	10	11	13	15	17	18	21	23	27	32	7	8	9	10	11	16	18	20	22	24
40-44	160-179	7	8	9	11	13	13	15	17	20	24	5	6	7	7	8	12	13	15	17	19
40-44	140-159	5	6	7	8	9	10	11	13	15	18	4	4	5	6	6	9	10	11	13	15
40-44	120-139	4	4	5	6	7	7	8	9	11	14	3	3	4	4	5	7	8	9	10	11
40-44	<120	3	3	4	4	5	5	6	7	8	10	2	3	3	3	4	5	6	7	8	9

Total cholesterol (mmol/l)

WHO cardiovascular disease risk non-laboratory based charts

Eastern Sub-Saharan Africa

Burundi, Comoros, Djibouti, Eritrea, Ethiopia, Kenya, Madagascar, Mozambique, Malawi, Rwanda, Somalia, Tanzania, Uganda, Zambia

Risk Level: <5% | 5% to <10% | 10% to <20% | 20% to <30% | ≥30%

Non-laboratory based risk chart

Age (years)	SBP (mmHg)	Men Non-smoker <20	Men Non-smoker 20-24	Men Non-smoker 25-29	Men Non-smoker 30-35	Men Non-smoker ≥ 35	Men Smoker <20	Men Smoker 20-24	Men Smoker 25-29	Men Smoker 30-35	Men Smoker ≥ 35	Women Non-smoker <20	Women Non-smoker 20-24	Women Non-smoker 25-29	Women Non-smoker 30-35	Women Non-smoker ≥ 35	Women Smoker <20	Women Smoker 20-24	Women Smoker 25-29	Women Smoker 30-35	Women Smoker ≥ 35
70-74	≥180	24	25	27	29	30	30	32	34	36	38	18	18	19	20	20	25	26	26	27	28
	160-179	20	21	22	24	25	25	26	28	30	32	15	15	16	16	17	21	22	22	23	24
	140-159	16	17	18	20	21	21	22	23	25	27	12	13	13	14	14	18	18	19	19	20
	120-139	13	14	15	16	17	17	18	19	21	22	10	11	11	11	12	15	15	16	16	17
	<120	11	11	12	13	14	14	15	16	17	18	9	9	9	10	10	12	13	13	14	14
65-69	≥180	19	20	22	23	25	25	27	29	31	34	14	14	15	16	16	21	22	23	24	24
	160-179	15	16	17	19	20	20	22	24	26	28	11	12	12	13	13	17	18	19	20	20
	140-159	12	13	14	15	16	16	18	19	21	23	9	10	10	10	11	14	15	15	16	17
	120-139	9	10	11	12	13	13	14	15	17	18	8	8	8	8	9	12	12	13	13	14
	<120	8	8	9	10	11	10	11	12	14	15	6	6	7	7	7	10	10	10	11	11
60-64	≥180	14	16	17	19	21	21	23	25	28	30	11	11	12	12	13	18	19	20	20	21
	160-179	11	12	14	15	16	17	18	20	22	24	9	9	9	10	10	15	15	16	16	17
	140-159	9	10	11	12	13	13	14	16	17	19	7	7	7	8	8	12	12	13	13	14
	120-139	7	7	8	9	10	10	11	12	14	15	5	6	6	6	6	9	10	10	11	11
	<120	5	6	6	7	8	8	9	10	11	12	4	4	5	5	5	7	8	8	8	9
55-59	≥180	11	12	14	15	17	17	19	22	24	27	9	9	9	10	10	15	16	17	18	19
	160-179	8	9	11	12	13	13	15	17	19	21	7	7	7	8	8	12	13	13	14	15
	140-159	6	7	8	9	10	10	11	13	15	16	5	5	6	6	6	9	10	10	11	11
	120-139	5	5	6	7	8	8	9	10	11	13	4	4	4	4	5	7	8	8	8	9
	<120	4	4	5	5	6	6	7	8	9	10	3	3	3	3	4	6	6	6	7	7
50-54	≥180	9	10	11	12	14	14	16	18	21	24	7	7	7	8	8	13	14	14	15	16
	160-179	6	7	8	9	11	11	12	14	16	18	5	5	5	6	6	10	10	11	12	12
	140-159	5	5	6	7	8	8	9	11	12	14	4	4	4	4	5	8	8	8	9	9
	120-139	3	4	4	5	6	6	7	8	9	10	3	3	3	3	3	6	6	6	7	7
	<120	3	3	3	4	4	4	5	6	7	8	2	2	2	2	3	4	5	5	5	6
45-49	≥180	7	8	9	10	11	12	14	16	18	21	5	5	6	6	6	11	12	12	13	14
	160-179	5	5	6	7	8	9	10	12	14	16	4	4	4	4	5	8	9	9	10	10
	140-159	3	4	5	5	6	6	7	9	10	12	3	3	3	3	3	6	6	7	7	8
	120-139	2	3	3	4	4	5	5	6	7	9	2	2	2	2	3	4	5	5	5	6
	<120	2	2	2	3	3	3	4	5	5	6	1	2	2	2	2	3	4	4	4	4
40-44	≥180	5	6	7	8	9	10	11	13	16	19	4	4	4	5	5	9	10	11	11	12
	160-179	4	4	5	6	7	7	8	10	11	14	3	3	3	3	4	7	7	8	8	9
	140-159	2	3	3	4	5	5	6	7	8	10	2	2	2	2	3	5	5	6	6	6
	120-139	2	2	2	3	3	3	4	5	6	7	1	2	2	2	2	3	4	4	4	5
	<120	1	1	2	2	2	2	3	4	4	5	1	1	1	1	1	3	3	3	3	3

Body mass index (kg/m^2)
